# Enzyme-free synthesis of cyclic single-stranded DNA constructs containing a single triazole, amide or phosphoramidate backbone linkage and their use as templates for rolling circle amplification and nanoflower formation†
†Electronic supplementary information (ESI) available: Experimental protocols, mass spectra, and additional gel electrophoresis results and SEM images. See DOI: 10.1039/c8sc02952k


**DOI:** 10.1039/c8sc02952k

**Published:** 2018-08-24

**Authors:** Jinfeng Chen, Ysobel R. Baker, Asha Brown, Afaf H. El-Sagheer, Tom Brown

**Affiliations:** a Chemistry Research Laboratory , University of Oxford , Oxford , OX1 3TA , UK . Email: tom.brown@chem.ox.ac.uk; b ATDBio , Magdalen Centre , Oxford Science Park , Oxford , OX4 4GA , UK; c Chemistry Branch , Department of Science and Mathematics , Suez University , Suez 43721 , Egypt

## Abstract

Cyclic oligonucleotides are valuable targets with a broad range of potential applications spanning molecular biology and nanotechnology. Of particular importance is their role as templates in the rolling circle amplification (RCA) reaction. We describe three different chemical cyclisation methods for the preparation of single-stranded cyclic DNA constructs. These chemical cyclisation reactions are cheaper to carry out than the enzymatic reaction, and more amenable to preparative scale purification and characterisation of the cyclic product. They can also be performed under denaturing conditions and are therefore particularly valuable for cyclic DNA templates that contain secondary structures. The resulting single-stranded cyclic DNA constructs contain a single non-canonical backbone linkage at the ligation point (triazole, amide or phosphoramidate). They were compared to unmodified cyclic DNA in rolling circle amplification reactions using φ-29 and Bst 2.0 DNA polymerase enzymes. The cyclic templates containing a phosphoramidate linkage were particularly well tolerated by φ-29 polymerase, consistently performing as well in RCA as the unmodified DNA controls. Moreover, these phosphoramidate-modified cyclic constructs can be readily produced in oligonucleotide synthesis facilities from commercially available precursors. Phosphoramidate ligation therefore holds promise as a practical, scalable method for the synthesis of fully biocompatible cyclic RCA templates. The triazole-modified cyclic templates generally gave lower and more variable yields of RCA products, a significant proportion of which were double-stranded, while the performances of the templates containing an amide linkage lie in between those of the phosphoramidate- and triazole-containing templates.

## Abstract 2

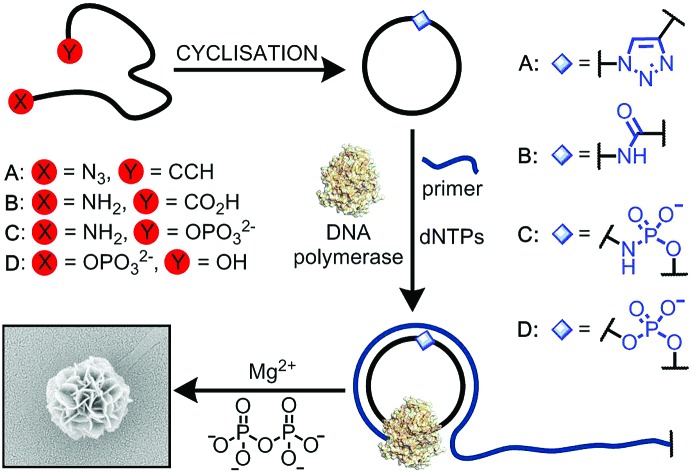
Three different chemical cyclisation reactions yield biocompatible cyclic oligonucleotide templates for use in RCA and DNA nanoflower formation.

## Introduction

**Fig. 1:**
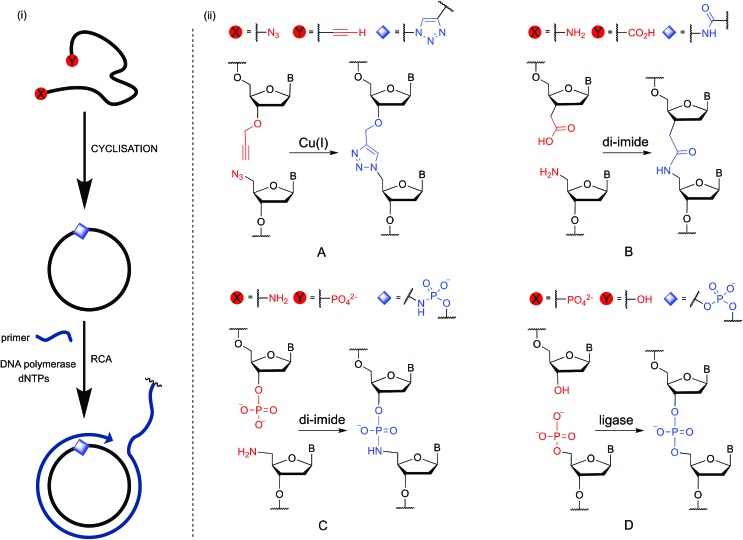
(i) Cyclisation of single-stranded DNA by chemical ligation methods and rolling circle amplification through the non-canonical linkage. (ii) Details of the chemical and enzymatic ligation strategies investigated in this study: (A) formation of an artificial triazole backbone linkage *via* the Cu(i)-catalysed azide–alkyne cycloaddition (CuAAC) reaction; (B) di-imide-mediated formation of an artificial amide backbone linkage; (C) di-imide-mediated formation of an artificial phosphoramidate backbone linkage; (D) enzyme-catalysed formation of a natural phosphate backbone linkage. B = nucleobase.

Rolling circle amplification (RCA) is an isothermal enzymatic technique that is widely used to make very long single-stranded DNA and RNA.^1^,^2^ The product contains a specific, tandem-repeating sequence which is encoded by the complementary cyclic template.^3^,^4^ This technique has been harnessed as a simple and powerful method of signal amplification in the development of sensitive detection methods for a variety of nucleic acid, protein, cellular and small molecule targets for diagnostic, biosensing and genotyping purposes.^5^–^14^ RCA has also attracted widespread interest as a tool in the synthesis of functional DNA nanomaterials^15^ including origami,^16^ nanosprings,^17^ nanotubes,^18^ templating nanoscaffolds,^19^,^20^ hydrogels^21^,^22^ and DNA nanoflowers.^23^ Proposed future applications of these materials include biosensors, targeted imaging and drug delivery agents,^24^ and components of nanoscale computers and electronic circuits. An RCA reaction requires four components: a circular DNA template, a short DNA primer, a polymerase enzyme and deoxynucleoside triphosphates (dNTPs). The cyclic template is most commonly obtained *via* an enzyme-catalysed cyclisation of a linear DNA strand using DNA ligase enzymes such as T4 ligase or CircLigase. The efficiency of the cyclisation reaction is sensitive to the length of the linear DNA precursor, and is inefficient if the nucleobase sequence leads to secondary structures.^25^,^26^ In addition, the scale on which the cyclisation reaction can be carried out is limited by the cost of the enzyme. In principle these limitations could be circumvented by the use of chemical ligation (Fig. 1), which is readily scalable, and would allow the use of a wide variety of buffers, including those in which DNA structures are denatured. However, the creation of a canonical phosphodiester linkage by chemical methods in aqueous media is inherently difficult because the nucleophilicity of the attacking hydroxyl group is similar to that of the surrounding water molecules, which are present in great excess. Although chemical methods for carrying out this transformation have been reported, such methods often rely on the use of acutely toxic reagents such as cyanogen bromide.^27^ For these reasons artificial DNA backbones which are less challenging to form chemically are an attractive alternative.^28^ Prevalent amongst these are the triazole,^29^ amide^30^,^31^ and phosphoramidate^32^–^34^ linkages (Fig. 1(ii)). When incorporated into linear oligonucleotides these phosphodiester backbone analogues can be read through by DNA polymerases during linear copying and the polymerase chain reaction (PCR), with accurate transfer of genetic information.^28^,^32^,^35^ They could potentially be incorporated into cyclic RCA templates provided that they can also be read through by the specific DNA polymerases that are compatible with efficient RCA. However, the use of chemically modified templates in RCA is particularly demanding because it requires the polymerase to accurately read through the modified linkage repeatedly in order to generate very long amplification products. In contrast, during PCR the polymerase needs only to copy the chemically modified template once during the first cycle, after which the reaction can proceed in an exponential manner, with the enzyme almost exclusively copying backbone-unmodified products in all subsequent cycles. Given the widespread importance of the RCA reaction, we undertook to study the cyclisation of DNA using chemical approaches and to examine the suitability of the chemically modified cyclic products as templates during RCA. We then demonstrated that RCA of the cyclic products can be used to prepare DNA nanoflowers.^23^,^36^–^41^ Finally, we compared the size distribution, morphologies and extent of DNA loading of the DNA nanoflowers assembled from the RCA products of the chemically modified templates.

## Results and discussion

### Choice of chemical linkages and template sequences

In the present study we investigate RCA of cyclic oligonucleotides containing a single triazole, amide or phosphoramidate analogue of the DNA phosphodiester backbone. All three modifications have been previously used in PCR amplification of linear DNA.^28^,^32^,^35^,^42^–^45^ Of these, the triazole linkage (Fig. 1(ii)A) is the most thoroughly studied artificial DNA backbone.^29^ It can be read through by both DNA and RNA polymerases and has been used in the construction of genes by chemical ligation. Importantly, DNA sequencing experiments did not reveal any increase in the level of mutations observed for cells transformed with the triazole-modified gene compared to those transformed with the canonical analogue.^35^ There are two reports of cyclisation of linear DNA that contains 5′-azide and 3′-alkyne followed by RCA of the cyclic template. The first one, from our laboratory,^45^ shows that cyclisation and subsequent RCA of a 100-mer DNA sequence designed to be free of secondary structure is possible, despite the presence of the 1,4-triazole linkage. The second study used a triazole-containing DNA template in an RCA-based fluorogenic assay for microRNA, but the authors did not compare the efficiency of the RCA reaction with that of an unmodified template.^46^ In this work we also investigate amide and phosphoramidate chemical linkages (Fig. 1(ii)B and C), both of which can be formed by di-imide coupling chemistry. The phosphoramidate linkage is of particular interest as it closely mimics the steric and electronic properties of the canonical phosphodiester backbone.

In order to study the above three non-canonical DNA linkages in RCA we designed three cyclic templates of differing base composition, all of which are predicted to incorporate secondary structures, and two of which include cancer cell surface targeting aptamer sequences (oligonucleotide sequences are given in Table 1, and the predicted structures are shown in Fig. S1, ESI†). Template **1** is based on a randomly generated 50-mer sequence with approximately 50% GC content; template **2** incorporates a dumbbell-shaped double hairpin structure and serves as a template for an aptamer sequence which targets the mucin 1 (MUC1) glycoprotein^47^—an important class of tumour surface marker that is overexpressed on a range of epithelial cancer cells; template **3** is a cytosine-rich sequence encoding a complementary trimeric G quadruplex aptamer which recognises the human epidermal growth factor receptor 2, a major therapeutic target in human cancer treatment.^48^ All four versions of each template were made: an unmodified control containing a natural phosphodiester (PO_4_) backbone and analogues containing triazole (Tz), amide (Am) and phosphoramidate (PA) linkages at the point of cyclisation.‡
‡Where necessary slight modifications were made to the ordering of the terminal nucleobases in order to make the sequences compatible with the chemistry used to introduce the required amino, azido or alkyne functionality at the 5′ and 3′ ends. Each phosphoramidate sequence contains 5′-amino dT; each amide sequence contains both 5′-amino dT and 3′-carboxy dT; and each triazole sequence contains 5′-azido dT and 3′-propargyl dC. See the ESI† for further details of the oligonucleotide synthesis chemistry. In addition we prepared the unmodified and triazole-modified cyclic templates **4**_PO_4_ and **4**_Tz, which incorporate a palindromic sequence. The RCA product of **4**_PO_4_ has been reported to assemble into a three-dimensional interwoven ‘nanoclew’ structure that can be used to deliver anticancer drugs.^39^ Following cyclisation of the linear sequences all of the cyclic templates were purified then subjected to RCA using φ-29, a highly processive polymerase with strand displacement activity.^49^

**Table 1:** Sequences (5′ → 3′) of the cyclic template oligonucleotides used in this study

Cyclic template	Sequence of linear oligonucleotide precursor^*a*^	Number of bases
**1**_PO_4_	^P^TCCCAATTGGGTACGCAGTACCCACAGCAGATGTGACTGTGAATCGTGAC	50
**1**_Tz	^Z^TCCCAATTGGGTACGCAGTACCCACAGCAGATGTGACTGTGAATCGTGAC^K^	50
**1**_Am	^M^TCCAATTGGGTACGCAGTACCCACAGCAGATGTGACTGTGAATCGTGACT^X^	50
**1**_PA	^M^TCCCAATTGGGTACGCAGTACCCACAGCAGATGTGACTGTGAATCGTGAC^P^	50
**2**_PO_4_	^P^TGTCGTTTTACCCATGTGCTATAGCCACTACTGTCGTTTTACCCATGTGCTATAGCCACTAC	62
**2**_Tz	^Z^TGTCGTTTTACCCATGTGCTATAGCCACTACTGTCGTTTTACCCATGTGCTATAGCCACTAC^K^	62
**2**_Am	^M^TGTCGTTTTACCCATGTGCTATAGCCACTACTGTCGTTTTACCCATGTGCTATAGCCACTAT^X^	62
**2**_PA	^M^TGTCGTTTTACCCATGTGCTATAGCCACTACTGTCGTTTTACCCATGTGCTATAGCCACTAC^P^	62
**3**_PO_4_	^P^TTACCCCACACCGCTGCCCCCACACCGCTGCCCCCACACCGCTGCCTTAC	50
**3**_Tz	^Z^TTACCCCACACCGCTGCCCCCACACCGCTGCCCCCACACCGCTGCCTTAC^K^	50
**3**_Am	^M^TACCCCACACCGCTGCCCCCACACCGCTGCCCCCACACCGCTGCCTTACT^X^	50
**3**_PA	^M^TTACCCCACACCGCTGCCCCCACACCGCTGCCCCCACACCGCTGCCTTAC^P^	50
**4**_PO_4_	^P^GTTAATATTATTCGACGGGCCTGCTCGAGCTCGAGCTTGCATCGTGCAGCCGAAGCTTGCACGCGTGCTATTAAT	75
**4**_Tz	^Z^TTGCACGCGTGCTATTAATGTTAATATTATTCGACGGGCCTGCTCGAGCTCGAGCTTGCATCGTGCAGCCGAAG^Me^C^K^	75

^*a*^P = 5′-PO_4_; M = 5′-amine, X = 3′-carboxy, Z = 5′-azide, K = 3′-propargyl, ^Me^C = methyl cytosine.

### Cyclisation of linear oligonucleotides

**Fig. 2:**
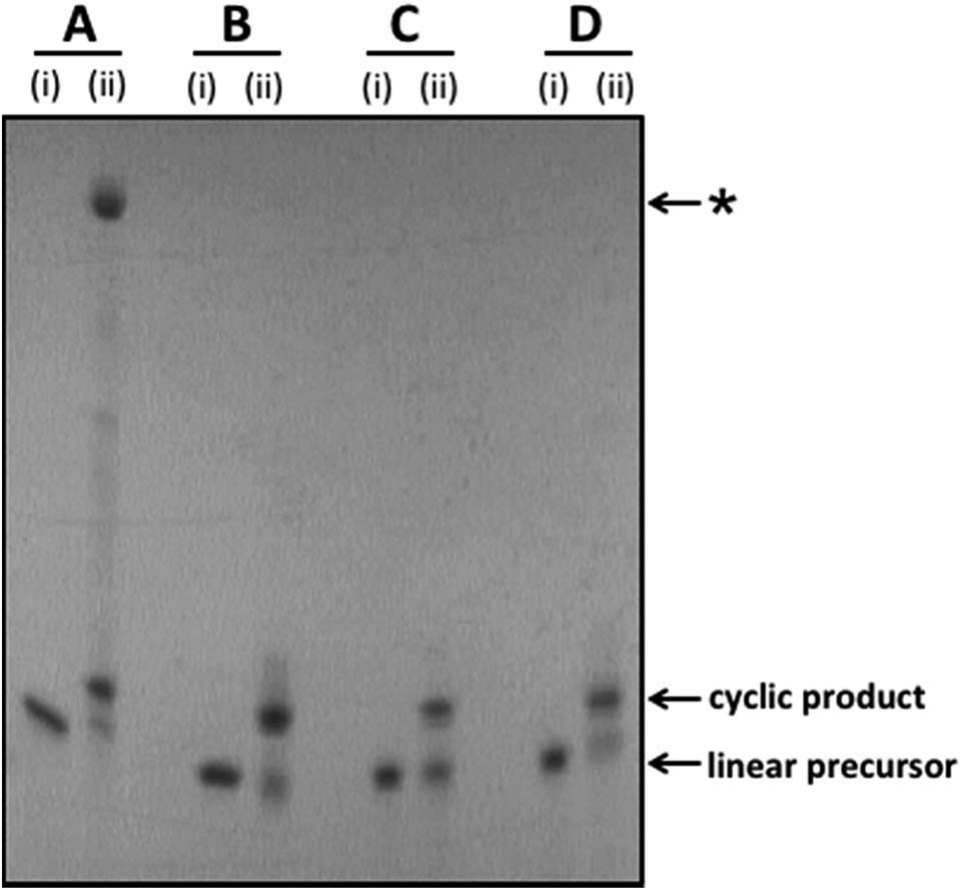
Representative examples of splint-mediated enzymatic and non-templated chemical cyclisation reactions to give the cyclic oligonucleotide templates **1**_PO_4_ (A), **1**_Tz (B), **1**_PA (C) and **1**_Am (D). The crude reaction mixtures were analysed by polyacrylamide gel electrophoresis on a 15% denaturing gel. Samples were imaged by UV shadowing. (A) Enzymatic cyclisation of linear unmodified 5′-phosphate/3′-hydroxyl oligonucleotide using T4 DNA ligase. (B) CuAAC-mediated cyclisation of linear 5′-azide/3′-alkyne oligonucleotide, introducing a triazole linkage. (C) Di-imide-mediated cyclisation of linear 5′-amine/3′-phosphate oligonucleotide, introducing a phosphoramidate linkage. (D) Di-imide-mediated cyclisation of linear 5′-amine/3′-carboxyl oligonucleotide, introducing an amide linkage. (i) = linear oligonucleotide substrate; (ii) = crude cyclisation reaction. *The major, slow-migrating side-product observed in the crude reaction mixture for the enzymatic ligation reaction is assigned as a cyclic dimer (molecular weight determined by UPLC-MS: 30 915 Da; calculated molecular weight for cyclic dimer: 30 912 Da; see Fig. S10, ESI†).

Linear oligonucleotides incorporating the required 5′-phosphate/3′-hydroxyl, 5′-azide/3′-alkyne, 5′-amine/3′-phosphate and 5′-amine/3′-carboxylate modifications were synthesised using automated solid phase phosphoramidite chemistry, as described in the ESI.† The linear unmodified control sequences were cyclised to give the cyclic templates **1**_PO_4_, **2**_PO_4_, **3**_PO_4_ and **4**_PO_4_ using T4 DNA ligase in the presence of a splint oligonucleotide. The splint oligonucleotides incorporate a region which is complementary to each end of the corresponding linear oligonucleotide and hence act as cyclisation templates by bringing the two reacting ends of the linear substrate into close proximity. Cyclisation of the chemically modified linear oligonucleotides was carried out under aqueous CuAAC or di-imide coupling conditions. In almost every case the linear precursor was successfully converted to the desired cyclic product with moderate to excellent efficiency.§
§The only exception is that the amide cyclisation reaction to give the cyclic template **3**_Am was inefficient, yielding a significant quantity of higher molecular weight side-products. Although we were able to isolate a sample of the cyclic template **3**_Am, it was found to contain impurities by HPLC mass spectrometry analysis (see Fig. S20, ESI†). Nonetheless, this sample was successfully used as a template in RCA, with promising results. The linear 5′-azide/3′-alkyne oligonucleotides were cyclised by treatment with CuSO_4_ in the presence of sodium ascorbate and tris-hydroxypropyltriazole (THPTA) in 0.2 M aqueous NaCl or denaturing organic/aqueous solvent mixtures. In all four cases the cyclisation reaction proceeded smoothly in the absence of a templating splint oligonucleotide. The 5′-amine/3′-phosphate oligonucleotides were cyclised by treatment with 1-ethyl-3-(3-dimethylaminopropyl)carbodiimide hydrochloride (EDC·HCl) and 2-(hydroxyethyl)imidazole in aqueous 0.2 M HEPES buffer (pH 7.2) using an adaptation of our previously reported phosphoramidate ligation method.^32^ Similarly the 5′-amine/3′-carboxylate were cyclised using EDC·HCl in combination with *N*-hydroxysuccinimide in aqueous NaCl or HEPES buffer, also using an adaptation of a previously reported method.^28^ The phosphoramidate- and amide-modified cyclic constructs **1**_PA and **1**_Am formed readily in the absence of a templating splint oligonucleotide; for the amide and phosphoramidate sequences **2**_PA, **2**_Am, **3**_PA and **3**_Am the cyclisation reactions were found to be less favourable under non-templating conditions and a splint oligonucleotide was therefore added in order to achieve more efficient cyclisation. Crude cyclisation reactions were analysed by polyacrylamide gel-electrophoresis (PAGE). Representative gel images for the template sequences **1**_PO_4_, **1**_PA, **1**_Tz and **1**_Am are shown in Fig. 2 (see Fig. S2–S9, ESI† for PAGE analysis of all other cyclisation reactions).

During the EDC-mediated cyclisation reactions to prepare the phosphoramidate-modified templates we observed the formation of adducts of the cyclic products with molecular weights which are 155 mass units higher than those for the expected products by UPLC-mass spectrometry. This is consistent with the chemical addition of EDC to the oligonucleotides. Similar observations have been documented previously: the adducts are proposed to arise from a chemical reaction between the di-imide coupling reagent and the imide group of guanine and thymine DNA bases.^50^–^54^ In support of this we did not observe formation of these adducts upon exposure of poly-adenine and poly-cytosine DNA sequences to 0.1 M EDC in 0.2 M HEPES buffer (pH 7.2) at room temperature, while in contrast a significant degree of EDC adduct formation was observed when the same experiment was performed with sequences containing guanine and thymine bases. Pleasingly it was possible to reverse this unwanted side-reaction by gently heating the crude product mixtures in 0.1 M NaOH after performing the phosphoramidate cyclisation reactions. Interestingly, we did not observe any evidence of the formation of the same EDC adducts during the reactions to prepare the amide-modified cyclic constructs, which were carried out under slightly different conditions and required a lower concentration of the di-imide coupling agent.

All of the modified and unmodified cyclic products were readily purified by PAGE. For the splint-mediated cyclisation reactions it was necessary to use denaturing PAGE conditions during purification, while for the non-templated reactions either denaturing or non-denaturing conditions were selected, depending on which gave better separation between the linear oligonucleotide precursor and cyclic oligonucleotide product. Full details of the cyclisation and purification protocols along with analytical data for all of the cyclic constructs are provided as ESI (Fig. S10–S23, ESI†).

**Fig. 3:**
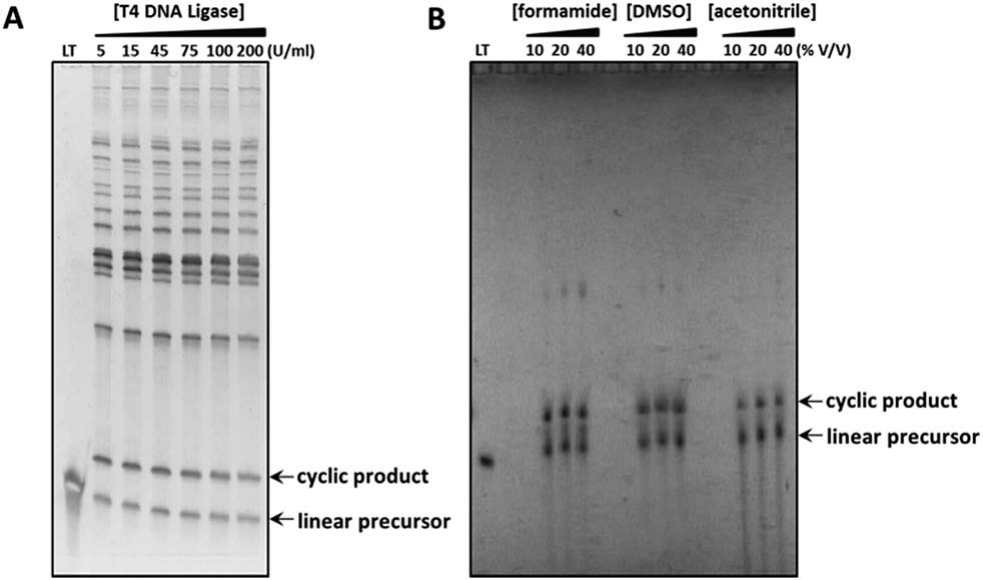
Comparison of the enzymatic and CuAAC-mediated cyclisations used to prepare the cyclic constructs **4**_PO_4_ and **4**_Tz respectively, whose sequences incorporate complex secondary structure. The crude reaction products were analysed using 8% denaturing polyacrylamide gels, which were visualised by post-staining with SYBR Gold (A) or by UV-shadowing (B). (A) Splint-mediated enzymatic cyclisation of the linear oligonucleotide in the presence of an increasing concentration of T4 DNA ligase. LT: linear 5′-phosphate/3′-hydroxyl functionalised oligonucleotide substrate. (B) Non-templated CuAAC-mediated cyclisation of the linear oligonucleotide in the presence of denaturing organic solvents. LT: linear 5′-azide/3′-alkyne functionalised oligonucleotide precursor.

While the efficiency of each cyclisation method described above was observed to vary depending on the individual linear oligonucleotide sequence, it is noteworthy that the chemical ligation methods can offer significant improvements in cyclisation efficiency in cases where the more commonly used enzymatic approach proves problematic. For example, while the T4-DNA ligase-mediated cyclisation of the ‘nanoclew’ template **4**_PO_4_ was low-yielding, producing a number of higher molecular weight oligomeric side-products, the analogous CuAAC-mediated cyclisation reaction—when performed in the presence of denaturing organic solvents to disrupt secondary structure—proceeded more cleanly, generating fewer by-products (Fig. 3). The efficiency of this chemical cyclisation reaction appears comparable to that of the reported CircLigase II-mediated cyclisation of the same template;^39^ however, the chemical cyclisation approach is not limited in scale by the high cost associated with the CircLigase enzyme. The chemical ligation methods discussed here therefore provide a greatly expanded toolkit for the cyclisation of linear oligonucleotides, with the potential to carry out the reactions under varied conditions, in the presence of denaturing agents, at varying salt concentration and temperature, and with significantly reduced cost. In addition, chemical ligation reactions are more robust and more scalable than enzymatic reactions, with attendant enhancements in the ease of purification, isolation and characterisation of the cyclic products.

### Rolling circle amplification of the cyclic oligonucleotides

#### RCA using the φ-29 polymerase

**Fig. 4:**
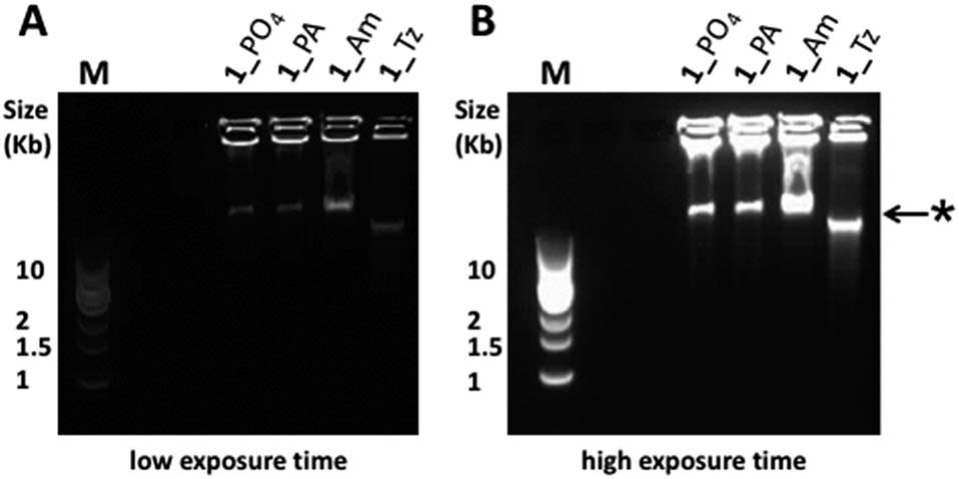
Agarose gel (0.8% agarose) analysis of the products formed during φ-29 polymerase catalysed RCA using cyclic templates **1**_PO_4_, **1**_Tz, **1**_Am and **1**_PA (8 hour amplification time). The RCA products were visualised by pre-casting the gel to contain a 0.5× concentration of SYBR® Gold nucleic acid gel stain. (A) Gel image using a short exposure time. (B) The same gel imaged using a longer exposure time, enabling visualisation of all products. M: 1 kb DNA ladder; *: this band is assigned to double-stranded DNA side-products.^55^

**Fig. 5:**
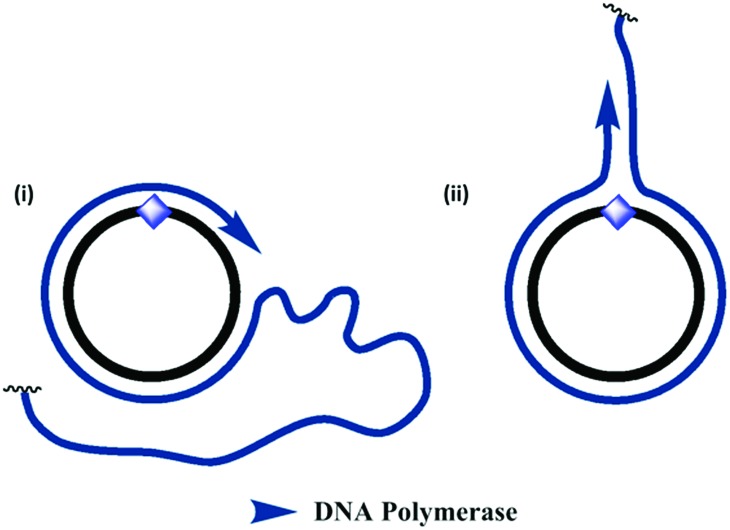
Proposed mechanism for the formation of double-stranded products during RCA of the triazole- and amide-modified cyclic templates. (i) In the normal RCA mechanism, the amplified single-stranded product is continuously displaced from the cyclic template, resulting in the formation of very long single-stranded products. (ii) An alternative mechanism allows the enzyme to dissociate from the cyclic template oligonucleotide and copy the emerging single-stranded product, resulting in the formation of double-stranded side-products. This process sequesters the cyclic template and inhibits RCA. This mechanism may be favoured in the presence of the unnatural triazole- and amide-backbone linkages, which the enzyme has greater difficulty in reading through.

For all fourteen cyclic templates, RCA was carried out using φ-29 polymerase, a 16 or 18 nucleobase primer, 2 mM dNTPs and 20 mM Mg^2+^ at 30 °C.¶
¶A lower Mg^2+^ concentration of 10 mM is recommended by the enzyme supplier. Our experiments indicated that, although RCA proceeds efficiently over a wide Mg^2+^ concentration range of 10–25 mM, the preparation on DNA-NFs is more sensitive to Mg^2+^ concentration: at 10 mM Mg^2+^ concentrations the extent of DNA loading is often negligible, whereas a higher 20 mM Mg^2+^ gives higher and more consistent levels of DNA loading. Taking this into account, we chose to use a 20 mM concentration of Mg^2+^ throughout our RCA experiments. Analysis of the product mixtures using agarose gel-electrophoresis indicated that in all cases the RCA reactions were successful (Fig. 4 and S24–S32, ESI†). However, there were significant differences in the quantities and size distributions of the products, which depended on the nature of the modified linkage in the cyclic template. Each reaction produced a detectable quantity of very slow-migrating products which were retarded in the wells of the gel and, in some cases, a second discrete band with higher gel mobility was also observed to form a substantial proportion of the product distribution (Fig. 4). By analogy with a recent literature report,^55^ this faster-running band was assigned to double-stranded products, while the material which is retarded in the wells is assigned to the expected very long single-stranded products. A possible mechanism for the formation of double-stranded side-products during RCA is illustrated in Fig. 5. It is noteworthy that the faster-running band assigned to double-stranded DNA side-products was not observed after RCA of the cyclic templates **3**_PO_4_, **3**_Am, **3**_PA and **3**_Tz, which encode a complementary trimeric G quadruplex aptamer sequence (Fig. S30–S32, ESI†). One possible explanation is that the mechanism of double-stranded DNA formation is suppressed by the tendency for the emerging RCA product to fold up into a G quadruplex secondary structure. The phosphoramidate modification gave very efficient RCA, consistently yielding product distributions which were indistinguishable from those of the unmodified templates (Fig. 4, S24, S27 and S30, ESI†). The amide-modified cyclic templates also appeared to be well-tolerated by the φ-29 polymerase, producing similar product distributions to the unmodified control sequences in all cases (Fig. 4, S25, S28 and S31, ESI†). In contrast, the triazole-containing templates gave weaker and more variable results. While the triazole-modified cyclic template **1**_Tz performed moderately in comparison to the unmodified and amide- and phosphoramidate-modified analogues, the cyclic templates **2**_Tz, **3**_Tz and **4**_Tz consistently produced much lower yields of very long, slow-migrating RCA products (Fig. 4; S26, S29 and S32, ESI†). The overall performance of the triazole-containing templates therefore appears to be compromised in comparison to those of the unmodified and phosphoramidate-containing analogues. This is a salient observation given that a recent publication uses a triazole-containing cyclic template with φ-29 in an RCA-based fluorescent switch-based diagnostic assay for microRNA detection.^46^ It is also interesting that during RCA of the amide- and triazole-modified templates **1**_Am, **1**_Tz and **2**_Am the proportion of double-stranded side-products appears to increase with extended amplification time. In contrast the relative proportions of the bands assigned to single- and double-stranded products respectively appear to stay approximately constant throughout the course of the RCA reactions for all of the unmodified and phosphoramidate-modified templates (Fig. S24–S28 and S30, ESI†).‖
‖We also investigated whether the formation of double-stranded amplification products could be suppressed by addition of single-stranded DNA binding protein to φ-29 mediated RCA reactions of the cyclic templates **1**_PO_4_ and **1**_PA, as previously described by Högberg and co-workers.^55^ However, when using our smaller, chemically modified templates we did not observe any significant differences in the product distributions of the RCA reactions carried out in the absence and presence of the single-stranded binding protein (see Fig. S47, ESI for further details†).


#### Probing the single-stranded RCA products with complementary fluorescent oligonucleotides

**Fig. 6:**
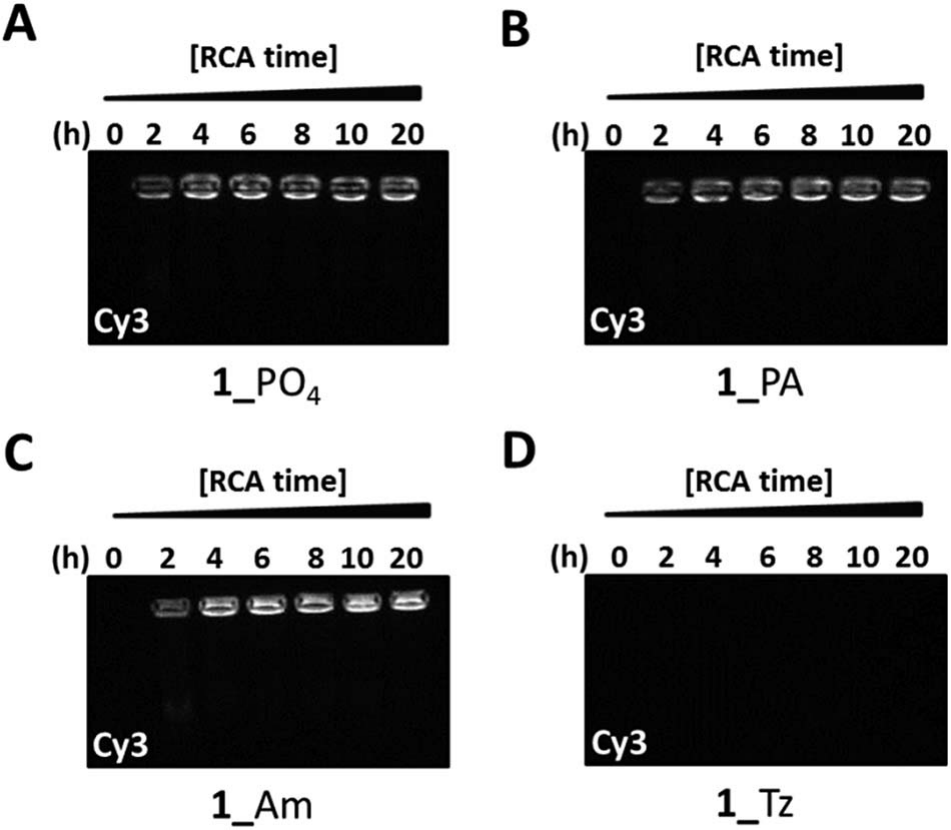
Probing the nature of the amplified products from φ-29-mediated RCA of the cyclic templates **1**_PO_4_ (A), **1**_PA (B), **1**_Am (C) and **1**_Tz (D) using a fluorescent probe hybridisation assay. Aliquots of the RCA reactions were stopped by heat inactivation at regular two hour intervals for a total period of 20 hours. A Cy3-labelled fluorescent probe with a sequence which is complementary to the RCA products was added to each aliquot and the mixtures were analysed by agarose gel electrophoresis (0.8% agarose). The gels were imaged under the Cy3 fluorescence channel. The gels were subsequently stained with SYBR Gold (Fig. S34, ESI†).

To obtain further information about the nature of the products from the RCA reactions on the modified phosphoramidate-, amide- and triazole-containing cyclic templates we developed a fluorescent probe assay to allow differentiation between single- and double-stranded RCA products by gel-electrophoresis. A single-stranded Cy3-labelled fluorescent hybridisation probe with a sequence which is complementary to those of the single stranded products obtained from RCA of the cyclic templates **1**_PO_4_, **1**_Tz, **1**_PA and **1**_Am was synthesised. In order to validate the assay, a scrambled version of the probe was also prepared, and we performed a preliminary control experiment using the complementary and scrambled versions of the hybridisation probe in order to confirm that the complementary probe binds specifically with the RCA product of the cyclic template **1**_PO_4_ through base paring (Fig. S33, ESI†). RCA of the four cyclic templates **1**_PO_4_, **1**_Tz, **1**_PA and **1**_Am was then performed using the φ-29-polymerase enzyme and individual RCA reactions were stopped by heat inactivation of the polymerase at two hour intervals over a twenty hour time period. The product mixtures were then incubated with the fluorescent hybridisation probe for two hours at room temperature before analysis using agarose gel electrophoresis. The gels were imaged with excitation at 520 nm under the Cy3 fluorescence channel of a Syngene G:BOX imager (Fig. 6). Finally, the same gels were post-stained with a solution of SYBR Gold, which can sensitively detect both double- and single-stranded DNA, and re-imaged with excitation at 302 nm (Fig. S34, ESI†). The results show that a strong fluorescence signal from the bound Cy3 labelled probe is detected after amplification of the unmodified, amide-modified and phosphoramidate-modified templates **1**_PO_4_, **1**_PA and **1**_Am. This demonstrates that the fluorescent probe has successfully hybridised to the RCA products, confirming their single-stranded nature. Conversely, after amplification of the triazole-modified template, no appreciable signal from the Cy3 fluorophore is detected, although some slow-migrating RCA products are weakly detected in the wells of the gel after post-staining with SYBR Gold. This suggests that the products of the RCA reaction using the triazole-modified template contain a significantly lower quantity of both single- and double-stranded DNA.

#### Probing the nature and quantity of the RCA products *via* a fluorometric plate-reading assay

**Fig. 7:**
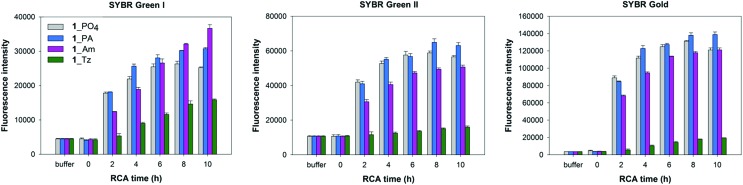
Fluorescence quantification of amplified DNA products from RCA reactions using the cyclic templates **1**_PO_4_, **1**_Am, **1**_PA and **1**_Tz and the φ-29 DNA polymerase over a 10 hour time course. EDTA was added to break down the precipitate releasing extended DNA prior to incubation with DNA binding dyes. Measurements are recorded in the presence of SYBR Gold, SYBR Green I and SYBR Green II fluorescent DNA binding dyes. Error bars represent the standard deviation of three measurements taken from a single RCA reaction. The reactions were also repeated in duplicate and the same trends were observed in both cases.

In order to corroborate the results from the gel-imaging we used a fluorometric plate-reading assay to allow a semi-quantitative comparison of the rates and total DNA yields of RCA reactions performed using φ-29 polymerase and the cyclic templates **1**_PO_4_, **1**_Tz, **1**_PA and **1**_Am over a 10 hour time course. After the desired time period the reactions were stopped by heat inactivation of the polymerase and then analysed, using three different DNA-binding dyes to provide a fluorescence signal: SYBR Gold, SYBR Green I and SYBR Green II. The intensities of the measured fluorescence signals are approximately proportional to the total concentrations of DNA in each reaction mixture, with some expected deviations as a result of the differing proportions of single- and double-stranded DNA amplification products. In agreement with the gel-imaging results, the reaction rates and product yields for the RCA reactions with the cyclic phosphoramidate-containing template **1**_PA are essentially the same as those for the unmodified cyclic template **1**_PO_4_, and the cyclic amide template **1**_Am performs almost as well. The cyclic triazole template **1**_Tz is shown to be inferior: at each time-point the measured fluorescence intensities are weaker for the RCA reaction involving the triazole-containing template **1**_Tz than for those involving the other three templates, indicating that a lower quantity of DNA is produced when **1**_Tz is used as a substrate. By taking into account the differing selectivities of the three DNA binding dyes for double-stranded DNA over single-stranded DNA it also possible to draw inferences about the nature of the products. While all three dyes are known to bind both double- and single-stranded DNA to some extent, SYBR Green I has the most pronounced selectivity for double-stranded DNA over single-stranded DNA.^56^–^58^ The measured fluorescence intensities for the samples containing the triazole-modified template relative to those containing the phosphate and phosphoramidate templates are noticeably higher in the presence of SYBR Green I than in the presence of the SYBR Gold and SYBR Green II dyes (Fig. 7). Hence there is a strong indication that a significant amount of double-stranded DNA is produced in the RCA reaction on the triazole-containing template, which is consistent with the prior gel-imaging results. The same phenomenon is observed for the amide-modified template **1**_Am, but to a much smaller extent. This is probably because each time the polymerase encounters the modified linkage in the cyclic template, it can choose between processively reading through the modified linkage or dissociating from the cyclic template and instead copying the RCA product as it emerges, thereby producing double-stranded products (Fig. 5).^55^ Since the triazole linkages are seemingly more difficult/slower to read through, it is likely that the alternative mechanism is more strongly favoured in their presence, resulting in the formation of a higher proportion of double-stranded products which would be of shorter length. This process may also sequester the cyclic template, contributing to the lower overall yield of DNA product.

#### RCA using the Bst 2.0 DNA polymerase

**Fig. 8:**
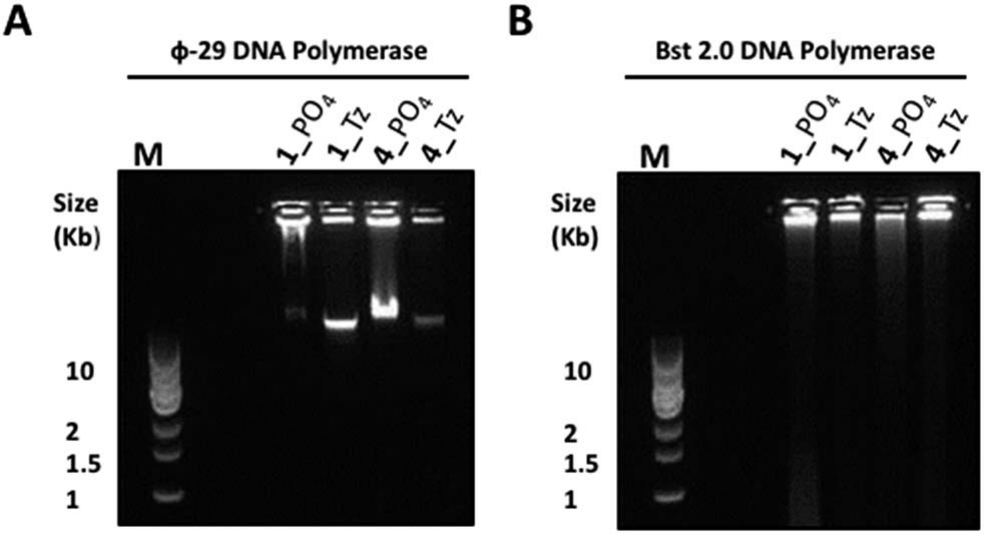
Agarose gel (0.8%) analysis of the product distributions from RCA of the triazole-modified cyclic templates **1**_Tz and **4**_Tz and the unmodified analogues **1**_PO_4_ and **4**_PO_4_ using φ-29 (A) and Bst 2.0 (B) polymerase enzymes after 20 hours. M: 1 kb DNA ladder; gels were pre-cast to contain a 0.5× concentrated solution of SYBR® Gold nucleic acid gel stain.

**Fig. 9:**
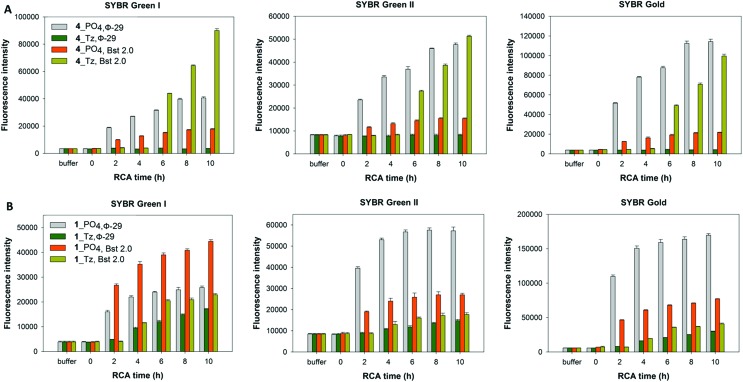
Fluorescence quantification of amplified DNA products from RCA reactions using the cyclic templates ((A) **4**_PO_4_, and **4**_Tz; (B) **1**_PO_4_, and **1**_Tz) and enzyme over a 10 hour time course. The performances of the φ-29 and Bst 2.0 DNA polymerases are compared. EDTA was added to break down the precipitate releasing extended DNA prior to incubation with DNA binding dyes. Measurements are recorded in the presence of SYBR Gold, SYBR Green I and SYBR Green II fluorescent DNA binding dyes. Error bars represent the standard deviation of three measurements taken from a single RCA reaction. The reactions were also repeated in duplicate and the same trends were observed in both cases.

In light of the disappointing performances of the triazole-containing templates in the φ-29-mediated RCA reactions, we also investigated RCA using the Bst 2.0 DNA polymerase for two of the cyclic triazole templates **1**_Tz and **4**_Tz and their unmodified analogues **1**_PO_4_ and **4**_PO_4_, in order to ascertain whether the triazole linkage can be read through more efficiently by this alternative polymerase. The Bst 2.0 enzyme was previously used for RCA of the cyclic template **4**_PO_4_ 39 and is known to efficiently incorporate modified dNTPs during RCA but its ability to read through modified backbone linkages has not yet been thoroughly tested.^59^,^60^ Agarose electrophoretic analysis of the RCA products indicated that, while the φ-29 enzyme again exhibited higher RCA efficiencies with the unmodified templates **1**_PO_4_ and **4**_PO_4_ compared to the triazole-containing analogues **1**_Tz and **4**_Tz, Bst 2.0 was able to process the triazole-modified templates more efficiently (Fig. 8). For example, reactions performed using the Bst 2.0 enzyme produced similar yields and product distributions for the triazole-modified and unmodified cyclic templates **1**_Tz and **1**_PO_4_, which are based on a randomly generated sequence (Fig. 8B). Moreover, for the nanoclew template sequences **4**_Tz and **4**_PO_4_ the Bst 2.0 DNA enzyme appeared to generate a significantly greater quantity of very long, slow-migrating RCA products from the triazole-containing template **4**_Tz than from the unmodified control **4**_PO_4_. Although unexpected, this result exemplifies the utility of having an available choice of chemical cyclisation methods in cases where the cyclisation and/or amplification of a specific canonical sequence proves to be inefficient. The RCA products from the reactions using the Bst 2.0 polymerase were also quantified *via* a fluorometric plate-reading assay, which corroborated the gel-imaging results. However it is notable that in all four reactions the Bst 2.0 enzyme generated a higher proportion of double-stranded products than the φ-29 enzyme (Fig. 9A and B)—reflecting the Bst enzyme's inferior processivity and strand displacement activity under the conditions used in our RCA experiments.

### Nanoflower formation

**Fig. 10:**
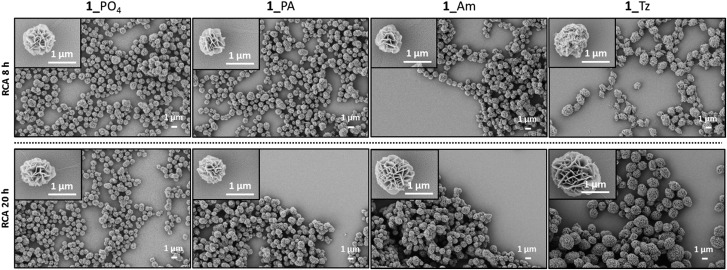
SEM images of the DNA-NFs generated from RCA of the cyclic templates **1**_PO_4_, **1**_PA, **1**_Am and **1**_Tz using the φ-29 polymerase in the presence of 20 mM Mg^2+^ after 8 hours and 20 hours.

**Fig. 11:**
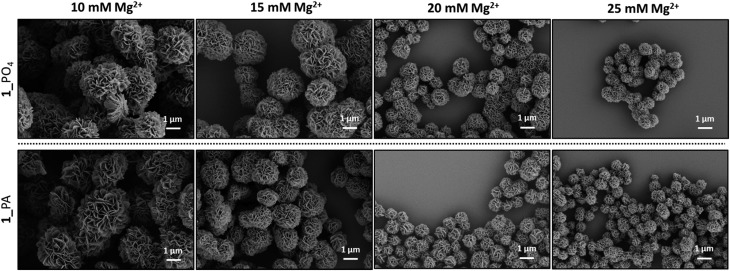
SEM images of the DNA-NFs formed from RCA of the cyclic templates **1**_PO_4_ (top) and **1**_PA (bottom) using the φ-29 polymerase in the presence of different concentrations of Mg^2+^. The RCA reactions were left for 20 hours and the Mg^2+^ concentrations ranged from 10–25 mM.

Long RCA products are known to spontaneously self-assemble into densely-packed DNA–inorganic hybrid nanoflower (DNA-NF) structures in the presence of a magnesium pyrophosphate co-precipitant, which is also generated as a by-product of the RCA reaction. The DNA-NF structures are thought to comprise an inorganic magnesium pyrophosphate core onto which the long single-stranded DNA RCA products are adsorbed. These nanoflower structures have several attractive features which are driving their investigation as potential diagnostic and therapeutic agents: they are easy to prepare; they are size-tuneable and biodegradable, yet resistant to digestion by exonuclease enzymes; they can be designed to incorporate a functional aptamer, DNAzyme, restriction enzyme, antisense or drug-loading sequence for targeted recognition and delivery; and they have been shown to be capable of cellular transfection.^23^,^36^–^41^ Scanning electron microscopy (SEM) experiments were used to map the size and morphology of the nanoflowers formed at different time points during the RCA reactions performed using the unmodified and modified cyclic templates. After time periods of eight and twenty hours aliquots of the reactions were removed and heated to inactivate the enzyme. The precipitated nanoflowers were then collected by centrifugation, thoroughly washed with deionised water and subjected to SEM imaging experiments. The extent of DNA loading was also examined by agarose gel electrophoresis (Fig. S35–S36, ESI†). The SEM images (Fig. 10 and 11) indicated that there was no significant difference in the size and morphology of the DNA nanoflowers generated from the four types of cyclic template, even though the triazole containing sequences are less efficient as RCA templates. In all cases DNA-NF structures with diameters of >1 μm and petal-like surface morphologies were formed in the RCA reaction mixture within eight hours, and their sizes were observed to have marginally increase after 20 hours. Variation of the Mg^2+^ concentration was found to have a significant effect on the particle size and DNA loading. At Mg^2+^ concentrations of ≤10 mM the particles were found to contain negligible quantities of DNA; as the Mg^2+^ concentration was increased from 10–25 mM the extent of DNA loading steadily increased, with a concomitant reduction in the size of the particles from ∼3 μm to ∼0.8 μm (Fig. 11 and S37–S46, ESI†). Although the reason for the contraction of the nanoparticles with increasing Mg^2+^ concentration is not immediately obvious, it is possible that this reflects an increased rate of nucleation at higher metal ion concentration, which might be expected to produce a higher number of particles with a smaller average diameter.

## Conclusions

We have shown that single-stranded DNA templates can be efficiently cyclised using three different chemical ligation methods. The resulting cyclic constructs contain a single unnatural triazole, amide or phosphoramidate linkage at the point of ligation. These chemical cyclisation methods offer several advantages over the more limited enzymatic approach: they are cheaper and easier to carry out, allowing for the large-scale synthesis and purification of the cyclic oligonucleotide products; they can also be performed under a wider variety of conditions, including denaturing ones, and are therefore particularly useful for cyclising linear oligonucleotides which contain problematic secondary structure. The chemical cyclisation methods described here therefore provide a versatile expanded toolkit for the preparation of cyclic oligonucleotides, which have an expansive range of potential applications across molecular biology^61^–^65^ and nanotechnology.^66^–^69^ Here we investigated the use of the chemically modified cyclic products as templates for the production of long single-stranded DNA concatemers *via* the highly demanding rolling circle amplification reaction, an important technique which is currently attracting widespread interest. The cyclic templates containing a non-canonical phosphoramidate backbone linkage performed particularly well during RCA—with overall yields, product distributions and reaction rates which were indistinguishable from those of the analogous unmodified templates. In contrast, the triazole-modified templates were less well tolerated by the φ-29 polymerase, showing a tendency to produce double-stranded DNA side-products and varying overall yields. The performance of the templates containing an amide modification falls in between those of the phosphoramidate and triazole linkages. Interestingly, for one of the cyclic template sequences, the Bst 2.0 polymerase enzyme was found to amplify the triazole-modified template with greater efficiency than the unmodified analogue, suggesting that different combinations of chemical ligation method and polymerase enzyme may find application where RCA proves challenging. Of all the cyclic templates investigated, the phosphoramidate-containing versions are the most straightforward to synthesise and could be produced in commercial DNA synthesis facilities from readily available precursors. We propose that phosphoramidate cyclisation is a practical, scalable and convenient method for the production of biocompatible cyclic DNA constructs for use in many applications, including RCA.

## Conflicts of interest

There are no conflicts to declare.

## Supplementary Material

Supplementary information
