# Supplementary material for: Enzyme-free synthesis of cyclic single-stranded DNA constructs containing a single triazole, amide or phosphoramidate backbone linkage and their use as templates for rolling circle amplification and nanoflower formation
Source: Chem Sci. 2018 Aug 24;9(42):8110–20. doi: 10.1039/c8sc02952k (PMC6238721; doi:10.1039/c8sc02952k)
Supplement: Supplementary file 1 [file SC-009-C8SC02952K-s001.pdf]

Electronic Supplementary Material (ESI) for Chemical Science.
This journal is © The Royal Society of Chemistry 2018

Supporting Information

Enzyme-free synthesis of cyclic single-stranded DNA constructs containing a single triazole, amide or phosphoramidate backbone linkage and their use as templates for rolling circle amplification and nanoflower formation

Jinfeng Chen,[a] Ysobel R. Baker,[a] Asha Brown,[b] Afaf H. El-Sagheer[a,c] and Tom Brown*[a]

[a.] Chemistry Research Laboratory, University of Oxford, Oxford, OX1 3TA, UK

[b.] ATDBio, Magdalen Centre, Oxford Science Park, Oxford, OX4 4GA, UK

[c.] Chemistry Branch, Department of Science and Mathematics, Suez University, Suez 43721, Egypt

*Tel: +44(0)1865 275413, e-mail: tom.brown@chem.ox.ac.uk

Contents

General experimental details 2
Oligonucleotide synthesis and purification 3
Table S1: Sequences of oligonucleotides used in this study 3
General methods for synthesis and purification of linear oligonucleotides 4
Synthesis of cyclic oligonucleotides 7
Predicted structures of circular templates 10
PAGE gels analyses of the crude cyclisation reaction products 11
Table S2: Oligonucleotide mass data 15
UPLC traces and mass spectra of the cyclic DNA templates used in this study 16
Rolling circle amplification 23
General procedure for agarose gel electrophoresis 23
General procedures for RCA reactions 23
Quantification of the RCA rate with Φ-29 DNA polymerase using fluorescent probes 23
RCA reaction catalysed using Φ-29 DNA polymerase with 10 mM Mg^{2+} and single stranded binding protein T4 gene 32 for cyclic templates (**1**_PO_4 and **1**_PA) 23
Measurement of fluorescence intensity of the RCA products stained with SYBR Green I, SYBR Green II and SYBR Gold 24
RCA reaction catalysed using Bst 2.0 DNA polymerase with 20 mM Mg^{2+} 24
DNA nanoflower experiments 24
Using the cyclic templates to prepare DNA-nanoflowers (DNA-NFs) 24
Comparing the effect of Mg^{2+} concentration on DNA loading of DNA-NFs 25
Comparing the effect of Mg^{2+} concentration on DNA-NF size 25

Images of agarose gels used to analyse the products from the RCA reactions 26
SEM images used to measure the particle size 36
References 38

General experimental details

All chemicals were purchased from Sigma-Aldrich unless otherwise specified and used without further purification. T4 DNA ligase was purchased from Promega. NxGen Φ-29 DNA polymerase was purchased from Lucigen. Bst 2.0 DNA polymerase, T4 gene 32 protein, deoxynucleotides (dNTPs) solution mix and Quick-Load® Purple 1 kb DNA ladder were purchased from New England Biolabs (UK). Scanning electron microscopy (SEM) consumables were purchased from Agar scientific. NAP-10 and NAP-25 columns were purchased from G.E. Healthcare Life Sciences. 96-well black, polystyrene, microplates were purchased from Greiner Bio-one. SYBR™ Gold Nucleic Acid Gel Stain, SYBR™ Green I Nucleic Acid Gel Stain and SYBR™ Green II RNA Gel Stain were purchased from ThermoFisher. 5X Nucleic acid sample loading buffer, used for non-denaturing polyacrylamide gel electrophoresis experiments, was purchased from Bio-rad. All RCA reactions were performed using a Bio-rad T100™ thermocycler.

Oligonucleotide synthesis and purification

Table S1: Sequences of oligonucleotides used in this study

a. Linear oligonucleotides used for cyclisation

Cyclic oligonucleotide code	Linear oligonucleotide precursor code	Sequence (5′–3′)
**1**_PO_4	ODN 1	^{P}TCCCAATTGGGTACGCAGTACCCACAGCAGATGTGACTGT GAATCGTGAC
**1**_Tz	ODN 2	^{Z}TCCCAATTGGGTACGCAGTACCCACAGCAGATGTGACTGT GAATCGTGAC^{K}
**1**_Am	ODN 3	^{M}TCCAATTGGGTACGCAGTACCCACAGCAGATGTGACTGTG AATCGTGACT^{X}
**1**_PA	ODN 4	^{M}TCCCAATTGGGTACGCAGTACCCACAGCAGATGTGACTGT GAATCGTGAC^{P}
**2**_PO_4	ODN 5	^{P}TGTCGTTTTACCCATGTGCTATAGCCACTACTGTCGTTTTA CCCATGTGCTATAGCCACTAC
**2**_Tz	ODN 6	^{Z}TGTCGTTTTACCCATGTGCTATAGCCACTACTGTCGTTTTA CCCATGTGCTATAGCCACTAC^{K}
**2**_Am	ODN 7	^{M}TGTCGTTTTACCCATGTGCTATAGCCACTACTGTCGTTTTA CCCATGTGCTATAGCCACTAT^{X}
**2**_PA	ODN 8	^{M}TGTCGTTTTACCCATGTGCTATAGCCACTACTGTCGTTTTA CCCATGTGCTATAGCCACTAC^{P}
**3**_PO_4	ODN 9	^{P}TTACCCCACACCGCTGCCCCCACACCGCTGCCCCCACAC CGCTGCCTTAC
**3**_Tz	ODN 10	^{Z}TTACCCCACACCGCTGCCCCCACACCGCTGCCCCCACACC GCTGCCTTAC^{K}
**3**_Am	ODN 11	^{M}TACCCCACACCGCTGCCCCCACACCGCTGCCCCCACACC GCTGCCTTACT^{X}
**3**_PA	ODN 12	^{M}TTACCCCACACCGCTGCCCCCACACCGCTGCCCCCACAC CGCTGCCTTAC^{P}
**4**_PO_4	ODN 13	^{P}GTTAATATTATTCGACGGGCCTGCTCGAGCTCGAGCTTGC ATCGTGCAGCCGAAGCTTGCACGCGTGCTATTAAT
**4**_Tz	ODN 14	^{Z}TTGCACGCGTGCTATTAATGTTAATATTATTCGACGGGCCT GCTCGAGCTCGAGCTTGCATCGTGCAGCCGAAG^{Me}C^{K}

P = PO_4, M = 5′-amine, X = 3′-carboxy, Z = 5′-azide, K = 3′-propargyl, ^{Me}C = methyl cytosine.

b. Splints and fluorescence probes used for cyclic templates

Oligonucleotide code	Sequence (5′-3′)
ODN 15 (Cyclisation splint for **1**_PO_4; RCA primer for **1**_PO_4, **1**_Tz and **1**_PA)	CAATTGGGAGTCACGATT
ODN 16 (Cyclisation splint for **2**_PO_4; RCA primer for **2**_PO_4, **2**_Tz and **2**_PA)	AAACGACAGTAGTGGC
ODN 17 (Cyclisation splint for **3**_PO_4; RCA primer for **3**_PO_4, **3**_Tz and **3**_PA)	TGGGGTAAGTAAGGCA
ODN 18 (Cyclisation splint for **4**_PO_4; RCA primer for **4**_PO_4 and 4_Tz)	ATAATATTAACATTAATAGCA
ODN 19 (Cyclisation splint and RCA primer for **1**_Am)	CCAATTGGAAGTCACGAT
ODN 20 (Cyclisation splint and RCA primer for **2**_Am)	AAAACGACAATAGTGGCT
ODN 21 (Cyclisation splint and RCA primer for **3**_Am)	TGTGGGGTAAGTAAGGCA
ODN 22 (Cyclisation splint for **2**_PA)	AAAACGACAGTAGTGGCT
ODN 23 (Cyclisation splint for **3**_PA)	GTGGGGTAAGTAAGGCAG
ODN 24 (fluorescence probe)	AATTGGGTACGCAGTACC-Cy3
ODN 25 (scrambled version of fluorescence probe ODN 24)	ATGTCAGACCTCGAAGGT-Cy3

General methods for synthesis and purification of linear oligonucleotides

Oligonucleotides were synthesised on an Applied Biosystems 394 automated DNA/RNA synthesiser using a standard 0.2 µmol or 1.0 µmol phosphoramidite cycle of acid-catalysed detritylation, coupling, capping and iodine oxidation. DNA phosphoramidites and reagents were purchased from Link Technologies, Applied Biosystems Ltd or Glen Research. Unless otherwise stated the syntheses were performed using 1000 Å controlled pore glass (CPG) solid supports with a particle size of 110 µm and nucleoside loading of 25–40 µm/g, purchased from Link technologies. Except where stated, standard β-cyanoethyl phosphoramidite monomers (benzoyl-dA, isobutylryl-dG, benzoyl-dC and dT) were used throughout. During the synthesis of the 3′-carboxy functionalised oligonucleotides, acetyl-dC was used in place of benzoyl-dC. Terminal 5′ amino dT modifications were added using the commercially available phosphoramidite monomer 5′-monomethoxytritylamino-2′-deoxythymidine,3′-[(2-cyanoethyl)-(*N*,*N*-diisopropyl)]-phosphoramidite, purchased from Glen research. Terminal 5′-azide functionality was introduced by post-synthetic modification of 5′-iodo dT containing oligonucleotides. Terminal 3′ phosphate modifications were incorporated using commercially available, pre-packed 3′ phosphate SynBase^{TM} CPG 1000/110 columns, purchased from Link technologies. Terminal 3′ alkyne functionality was introduced using a 3′ propargyl dC or 3′ propargyl 5-methyl dC functionalised CPG resin, prepared as described previously.(1) The coupling time for addition of standard A, G, C and T monomers was set to 60 seconds. For addition of modified monomers the coupling time was extended to 600 seconds. Stepwise coupling efficiencies and overall yields were monitored using the instrument's in-built automated trityl cation conductivity measurement facility.

Except where stated the oligonucleotides were cleaved from the solid support and deprotected by exposure to concentrated aqueous ammonia solution at room temperature (RT) for 60 min followed by heating in a sealed glass vial at 55 °C for 5 hours. The aqueous solution of ammonia was then removed by evaporation prior to oligonucleotide purification.

The oligonucleotides were purified by reverse-phase high performance liquid chromatography (RP-HPLC). All 5′ amino functionalised oligonucleotides were purified on a Shimadzu LC-20AP HPLC system with a Waters XBridge OST C_{18} column (particle size: 2.5 µm; pore size: 100 Å; column dimensions: 1.9 x 50 mm). The following elution buffers were used: buffer A: 0.1 M triethylammonium acetate$_{(aq)}$; buffer B: 0.1 M triethylammonium acetate$_{(aq)}$ containing 20% CH_3CN; gradient 30–70% buffer B over 9 minutes. The flow rate was 15 mL/min. The elution of oligonucleotides was monitored by UV absorption at 260 nm. The pure fractions were combined, evaporated and de-salted using a NAP-25 column followed by a NAP-10 column. All other oligonucleotides were purified on a Gilson system using a Luna 10 µm C8 100 Å pore Phenomenex 10 x 250 mm column with a gradient of acetonitrile in triethylammonium bicarbonate (TEAB) increasing from 0% to 50% buffer B over 20 min with a flow rate of 4 mL/min (buffer A: 0.1 M TEAB, pH 7.5, buffer B: 0.1 M TEAB, pH 7.5 with 50% acetonitrile). The elution of oligonucleotides was monitored by UV absorption at 298 nm. After HPLC purification, the oligonucleotides were freeze dried and then re-dissolved in water without the need for further de-salting.

All oligonucleotides were characterised by negative-mode UPLC-mass spectrometry using either a Bruker micrOTOF^{TM} II focus ESI-TOF mass spectrometer with an Acquity UPLC system, equipped with a Ethylene Bridged Hybrid (BEH) C_{18} column (Waters) or a Waters Xevo G2-XS QT of mass spectrometer with an Acquity UPLC system, equipped with an Acquity UPLC oligonucleotide BEH C_{18} column (particle size: 1.7 µm; pore size: 130 Å; column dimensions: 2.1 x 50 mm). Data were analysed using Waters MassLynx software or Waters UNIFI Scientific Information System software.

Further details of the syntheses of the chemically modified oligonucleotides are provided below.

Synthesis of 5′-phosphate oligonucleotides. Terminal 5′ phosphate modifications were added using a commercially available 2-[2-(4,4′-dimethoxytrityloxy)ethylsulfonyl]ethyl-(2-cyanoethyl)-(*N*,*N*-diisopropyl)-phosphoramidite, purchased from Link technologies, using an extended coupling time of 600 seconds. The oligonucleotides were cleaved from the solid support, deprotected and purified as described above.

Synthesis of 3′-alkyne-5′-azide oligonucleotides. 5′-O-(4,4′-Dimethoxytrityl)-3′-O-propargyl-deoxycytidine or 5′-O-(4,4′-dimethoxytrityl)-3′-O-propargyl-5-methyldeoxycytidine on polystyrene solid support, prepared as previously described,(1) was packed into a twist column (Glen Research) and used to synthesise the required sequences in the 3′- to 5′-direction (standard phosphoramidite oligonucleotide synthesis) with 5′-iodo dT as the final nucleotide. The 5′-iodo dT modification was

introduced using a commercially available 5′-iodo-2′-deoxythymidine, 3′-[(2-cyanoethyl)-(*N*,*N*-diisopropyl)]-phosphoramidite, purchased from Glen research. To convert the 5′-iodo dT to 5′-azido dT, sodium azide (20 mg) was suspended in dry DMF (1 ml) and heated at 70 °C for 20 min followed by cooling down to RT. The supernatant was taken up into a 1 mL syringe, passed back and forth through the column periodically, and then left at 55 °C for 5 h. The column was washed with DMF (3 X 1 mL) followed by acetonitrile (3 X 1 mL) and dried by the passage of a stream of argon gas. The resultant 5′-azide oligonucleotide was cleaved from the solid support, deprotected and purified as described above.

Synthesis of 5′-amino-3′-phosphate oligonucleotides. Oligonucleotides were synthesised on commercially available, pre-packed 3′ phosphate SynBase^{TM} CPG 1000/110 columns, purchased from Link technologies. The terminal 5′ amino dT modification was added using the commercially available phosphoramidite monomer 5′-monomethoxytritylamino-2′-deoxythymidine,3′-[(2-cyanoethyl)-(*N*,*N*-diisopropyl)]-phosphoramidite, purchased from Glen research, using an extended coupling time of 600 seconds. During addition of the final 5′ amino dT nucleotide the acetic anhydride capping step was omitted from the synthesis cycle in order to avoid acetylation of the amino group. The oligonucleotide was cleaved from the solid support, deprotected and purified according to the general procedure described above.

Synthesis of 5′-amino-3′-carboxy oligonucleotides. In order to introduce the terminal 3′ carboxylic acid functionality, oligonucleotides were synthesised on a 3′ carboxy dT functionalised polystyrene resin, prepared as previously described.(2) During oligonucleotide synthesis the use of benzoyl-protected dC phosphoramidite was avoided in favour of acetyl-dC, because benzoyl dC is known to undergo a deamination side-reaction under the sodium hydroxide mediated deprotection conditions used in this procedure. The terminal 5′ amino dT modification was added using the commercially available phosphoramidite monomer 5′-monomethoxytritylamino-2′-deoxythymidine,3′-[(2-cyanoethyl)-(*N*,*N*-diisopropyl)]-phosphoramidite, purchased from Glen research, using an extended coupling time of 600 seconds. Prior to the addition of this final nucleotide the resin was treated with a 20% v/v solution of di-isopropylethylamine in acetonitrile for 15 minutes, washed three times with acetonitrile and dried under a stream of argon. During addition of the final 5′ amino dT nucleotide the acetic anhydride capping step was omitted from the synthesis cycle in order to avoid acetylation of the amino group. The resin was then treated with 0.5 mL of a solution 0.4 M of NaOH in MeOH:H_2O 4:1 v/v for six hours at RT. At this point 0.5 mL of a solution 0.4 M of NaOH in MeOH:H_2O 1:4 v/v was added, to give a 0.4 M solution of NaOH in MeOH:H_2O 1:1 v/v. The mixture was allowed to stand at RT for a further 40 hours before being neutralized by addition of 1 mL of triethylammonium acetate buffer (1 M, pH 7.4), concentrated on a rotary evaporator and de-salted using a NAP-25 column. To ensure complete cleavage of all isobutyryl protecting groups, the product was taken up in concentrated aqueous ammonia and heated at 55 °C for 2–5 hours in a sealed glass vial. After concentration of the aqueous ammonia solution *in vacuo* the oligonucleotide was purified according to the general procedure described above.

Synthesis of cyclic oligonucleotides

Procedure for denaturing polyacrylamide gel electrophoresis (PAGE). The DNA samples were mixed with an equal volume of formamide, or with a 20% volume of 5X Nucleic Acid Sample Loading Buffer from Bio-Rad (pH 8, 50 mM Tris-HCl, 25% glycerol, 5 mM ethylenediaminetetraacetic acid (EDTA), 0.2% bromophenol blue, 0.2% xylene cyanole FF) and 7 M urea. Samples denatured by heating at 95 °C for 5 min. The mixture was cooled quickly on ice, and then loaded on denaturing PAGE gel (acrylamide:bisacrylamide 19:1, 40% acrylamide solution, 7 M urea, prepared in a 1X Tris-borate-EDTA (TBE) buffer (89 mM Tris; 89 mM boric acid; 2 mM EDTA)). The gel was run at either 20 W or 200 V for the desired time period (1–2.5 h) in 1X TBE buffer at RT. Gels were visualised using a G:Box gel documentation system (Syngene). The cyclised oligonucleotide bands were excised, crushed and soaked in MilliQ water (10-15 mL) overnight at 37 °C with shaking. After filtration and evaporation of the MilliQ water, the cyclised oligonucleotides were desalted using NAP-25, followed by a NAP-10 column. The concentration of cyclised oligonucleotides was measured by using a UV-Vis spectrophotometer (Cary, model 50 Bio, Varian, Australia).

Procedure for non-denaturing (PAGE). The DNA samples were mixed with 5X Nucleic Acid Sample Loading Buffer from Bio-Rad (4:1 v/v) (pH 8, 50 mM Tris-HCl, 25% glycerol, 5 mM EDTA, 0.2% bromophenol blue, 0.2% xylene cyanole FF) and loaded onto a non-denaturing PAGE gel (acrylamide:bisacrylamide 19:1, 40% acrylamide solution, prepared in a 1X TBE buffer). The gel was run at a constant voltage of 150 V in 1X TBE buffer at RT for the desired time length. Gels were visualised using a G:Box gel documentation system (Syngene). The cyclised oligonucleotide bands were excised, crushed and soaked in MilliQ water (10-15 mL) overnight at 37 °C with shaking. After filtration and evaporation of the MilliQ water, the cyclised oligonucleotides were desalted using NAP-25, followed by a NAP-10 column. The concentration of cyclised oligonucleotides was measured by using a UV-Vis spectrophotometer (Cary, model 50 Bio, Varian, Australia).

T4 DNA ligase-mediated cyclisations to give the cyclic oligonucelotides 1_PO_4, 2_PO_4 and 3_PO_4. A 5′-phosphorylated linear oligonucloetide (ODN 1, ODN 5 or ODN 9; 2.4 nmol, 1 eq) and a templating splint oligonucleotide (ODN 15, ODN 16 or ODN 17; 7.2 nmol, 3 eq) were annealed in 3.9 mL of 1X T4 DNA ligase buffer (30 mM Tris-HCl, pH 7.8 @ 25 °C, 10 mM $MgCl_2$, 10 mM DTT, and 1 mM ATP) by heating to 95 °C for 5 min, followed by cooling to RT at a rate of 0.5 °C/min. T4 DNA ligase (100 µL, 3U/µL) was then added to the solution which was incubated at RT overnight. The sample was heated to 70 °C for 15 min to denature the enzyme. The volume of reaction mixture was then reduced to 2.5 mL using a CentriVap centrifugal concentrator at 55 °C followed by desalting using NAP-25 column. The cyclised oligonucleotides was purified by denaturing PAGE (**1**_PO_4: 15% PAGE, **2**_PO_4: 8% PAGE and **3**_PO_4: 12% PAGE) as described above.

Preparation of the cyclic oligonucleotide 4_PO_4 using T4 DNA ligase. The 5′-phosphorylated linear oligonucleotide ODN 13 (2.4 nmol, 1 eq) and the templating splint oligonucleotide ODN 18 (4.8 nmol, 2

eq) were annealed in 3.9 mL of 1X T4 DNA ligase buffer (30 mM Tris-HCl, pH 7.8 @ 25 °C, 10 mM $MgCl_2$, 10 mM DTT, and 1 mM ATP) by heating to 95 °C for 5 min, followed by cooling to RT at a rate of 0.5 °C/min. T4 DNA ligase (100 µL, 3U/µL) was then added and the solution was incubated at RT overnight. The sample was heated to 70 °C for 15 minutes to denature the enzyme. The volume of reaction mixture was reduced to 2.5 mL using a CentriVap centrifugal concentrator at 55 °C followed by desalting using NAP-25 column. The cyclised oligonucleotides was purified by 8% denaturing PAGE as described above.

Preparation of Cu^I click catalyst solution. To prepare a solution of Cu^I click catalyst, tris-hydroxypropyltriazole ligand (THPTA) (3.6 mg) was dissolved into a solution of sodium ascorbate (12 µmol in MilliQ water, 24 µL) followed by degassing by bubbling with argon gas (~ 3 min). $CuSO_4.5H_2O$ (1.2 µmol in MilliQ water, 12 µL) was added and the solution was degassed again.

Preparation of the cyclic oligonucleotides 1_Tz, 2_Tz and 3_Tz containing triazole linkages through CuAAC 'click' ligation. Cu^I click catalyst solution (36 µL, prepared as described above) was added to 2.364 mL of the linear 5′-azide-3′-propargyl oligonucleotide (ODN 2, ODN 6 or ODN 10; 12 nmol in 0.2 M $NaCl_{(aq)}$,). The reaction mixture was kept at RT for 2.5 h after which reagents were removed by NAP-25 gel filtration. The volume of reaction mixture was concentrated using a CentriVap centrifugal concentrator at 55 °C. The cyclised DNA products were analysed and purified by denaturing PAGE (**1**_Tz: 15% PAGE, **2**_Tz: 8% PAGE and **3**_Tz: 12% PAGE) as described above.

Non-templated cyclisation to give the cyclic oligonucleotide 4_Tz containing a triazole linkage through CuAAC 'click' ligation under denaturing condition. 1.2 nmol of the linear 5′-azide-3′-propargyl oligonucleotide ODN 14 was first dissolved in 236 µL of the organic solvent/MilliQ water solution (9:1, 4:1 or 3:2 v/v of MilliQ water : formamide, DMSO or acetonitrile) and heated to 95 °C for 5 min followed by cooling rapidly on ice. A solution of Cu^I click catalyst (3.6 µL, prepared as described above) was added to the reaction, and the solution was kept at RT for 2.5 h after which reagents were removed by NAP-10 gel filtration. The volume of reaction mixture was concentrated using a CentriVap centrifugal concentrator at 55 °C. The cyclised DNA products were analysed and purified by 8% denaturing PAGE as described above.

Non-templated cyclisation to give the modified circular template 1_PA containing a phosphoramidate linkage. The linear 3′-phosphate/5′-amino dT oligonucleotide ODN 4 (10 nmol, 4 µM) was dissolved in 2.5 mL of 0.2 M HEPES buffer (pH 7.2). 1-(2-Hydroxyethyl)imidazole (0.25 mmol, 0.1 M) was added as a solid, followed by *N*-(3-dimethylaminopropyl)-*N*′-ethylcarbodiimide hydrochloride (EDC·HCl) (0.19 mmol, 0.075 M). The solution was allowed to stand at RT for 3 h. An additional portion of EDC·HCl (0.19 mmol) was then added and the solution was left at RT for a further 13 h, before removal of the small molecule reagents and buffer by gel-filtration, using a NAP-25 column, followed by a NAP-10 column. The crude product was taken up in 1 mL of 0.1 M $NaOH_{(aq)}$ and the solution was heated at 55 °C for 6 h, then cooled to RT and de-salted using a NAP-25 column followed

by a NAP-10 column. The crude product mixture was purified by non-denaturing 15% PAGE as described above.

Templated cyclisations to give the modified circular templates 2_PA and 3_PA containing a phosphoramidate linkage. An equimolar solution of the linear 3′-phosphate/5′-amino dT oligonucleotide (ODN 8 or ODN 12; 4 µM) and the splint oligonucleotide (ODN 22 or ODN 23; 4 µM) in 0.2 M HEPES buffer (pH 7.2) was heated to 95 °C for 5 min and then allowed to cool slowly to RT over approximately 2 h. 1-(2-Hydroxyethyl)imidazole and EDC·HCl were added as solids in one portion to give a 0.2 M concentration of both reagents. The reaction mixture was allowed to stand at RT for 15 h. The reagents and buffer were then removed by gel filtration using a NAP-25 column followed by a NAP-10 column. The crude product mixture was taken up in 1 mL 0.1 M $NaOH_{(aq)}$ and heated at 55 °C for 5 h. After cooling to RT the NaOH was removed using a NAP-25 column followed by a NAP-10 column and the product mixture was analysed and purified by denaturing PAGE (13% and 15% for **2**_PA and **3**_PA respectively) as described above.

Non-templated cyclisation to give the modified circular template 1_Am containing an amide linkage. The linear 3′-carboxy/5′-amino dT oligonucleotide ODN 3 (5 nmol) was dissolved in 1.0 mL of 0.125 M $NaCl_{(aq)}$. *N*-Hydroxysuccinimide (125 µL of a 25 mM solution in H_2O) and EDC·HCl (125 µL of a 100 mM solution in H_2O) were added. The reaction mixture was allowed to stand at RT for 2 h, before removal of reagents and salt using a NAP-25 column. The product mixture was purified by non-denaturing 15% PAGE as described above.

Templated cyclisations to give the modified circular template 2_Am containing an amide linkage. An equimolar solution of the linear 3′-carboxy/5′-amino dT oligonucleotide ODN 7 (1 nmol) and the splint oligonucleotide ODN 20 (1 nmol) in 0.2 mL of 0.125 M $NaCl_{(aq)}$ was heated to 90 °C and allowed to cool slowly to RT over approximately 2 h. *N*-Hydroxysuccinimide (25 µL of a 25 mM solution in H_2O) and EDC·HCl (25 µL of a 100 mM solution in H_2O) were added. The reaction mixture was allowed to stand at RT for 2 h before removal of reagents and salts using a NAP-25 column. The product mixture was analysed and purified by denaturing 13% PAGE as described above.

Templated cyclisation to give the modified circular template 3_Am containing an amide linkage. An attempt to prepare the cyclic template **3**_Am under the conditions described above for the preparation for **2**_Am was unsuccessful. In order to obtain the cyclic template **3**_Am the procedure was modified as follows. An equimolar solution of the linear 3′-carboxy/5′-amino dT oligonucleotide ODN 11 (5 nmol) and the splint oligonucleotide ODN 21 (5 nmol) in 1.0 mL of 0.2 M HEPES buffer (pH 7.2) was heated to 90 ºC and allowed to cool slowly to RT over approximately 2 h. *N*-Hydroxysuccinimide (125 µL of a 25 mM solution in H_2O) and EDC·HCl (125 µL of a 100 mM solution in H_2O) were added. The reaction mixture was allowed to stand at RT for 18 h, before removal of the reagent and buffer using a NAP-25 column. The product mixture was analysed and purified by denaturing 15% PAGE as described above. UPLC-MS analysis of the isolated product indicated that it contained inseparable impurities with

masses 71 and 142 units higher than that of the correct product. Although the identities of these side-products are uncertain and still under investigation, they are tentatively assigned as either partially hydrolysed EDC adducts of the cyclic product, or incompletely deprotected analogues containing residual *N*-isobutyryl-functionalised guanine groups.

Predicted structures of circular templates

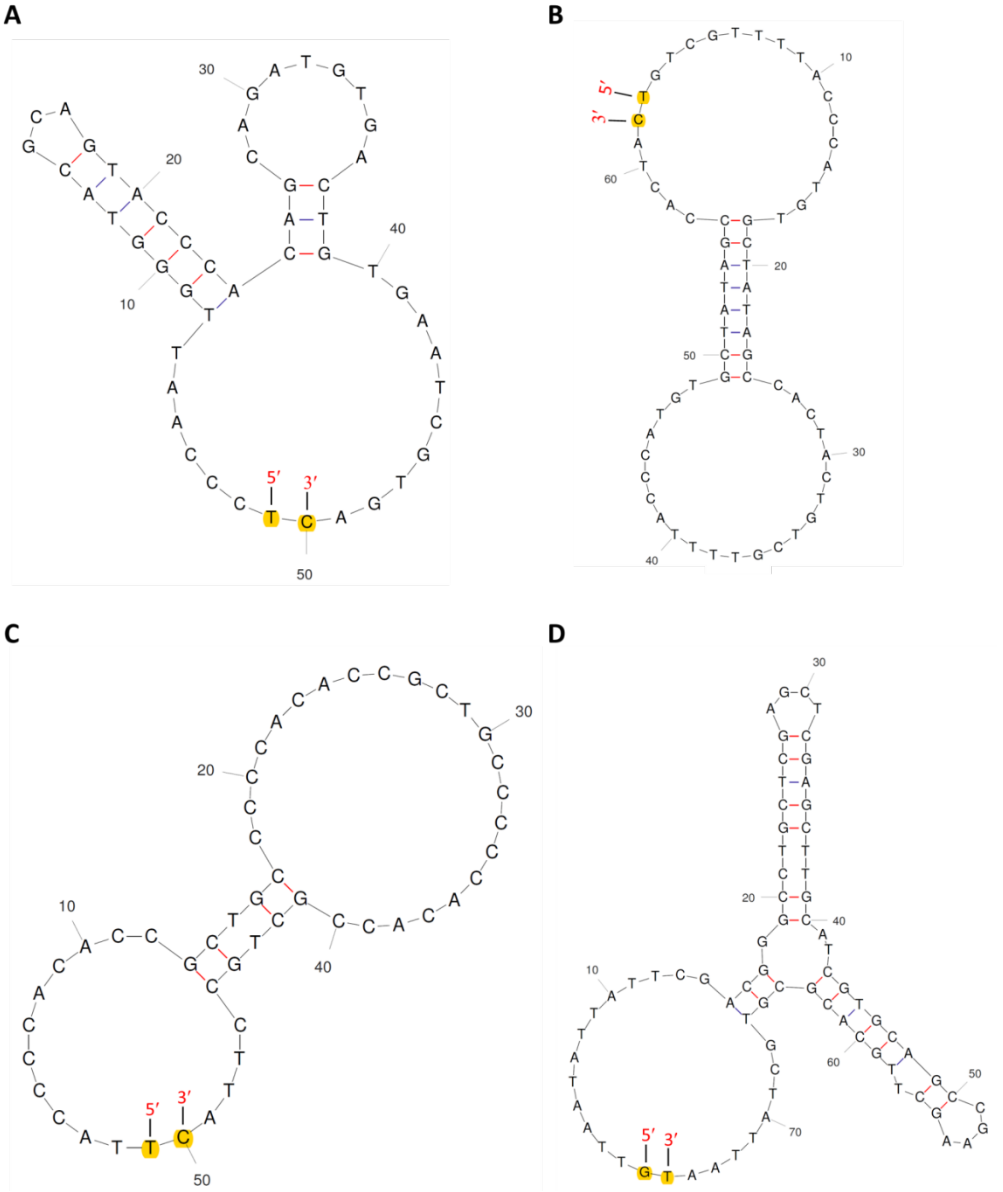

Figure S1. Predicted secondary structures of the circular templates used in this study. (A) template **1;** (B) template **2;** (C) template **3** and (D) template **4**. Structures were predicted using the Mfold web server software.(3) The highlighted bases indicate the point of ligation.

PAGE gels analyses of the crude cyclisation reaction products

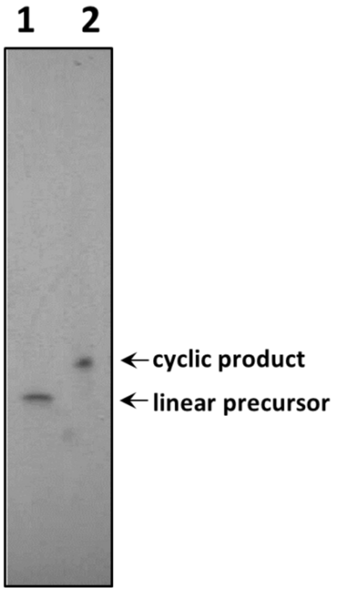

Figure S2. Splint-mediated enzymatic cyclisation of template (**2**_PO_4) using T4 DNA ligase. 8% denaturing PAGE gel analysis of 5′-phosphate/3′-hydroxyl oligonucleotide cyclisation to generate circular template with phosphodiester linkage. Lane 1: linear 5′-phosphate/3′-hydroxyl functionalised template; Lane 2: cyclisation reaction mixture.

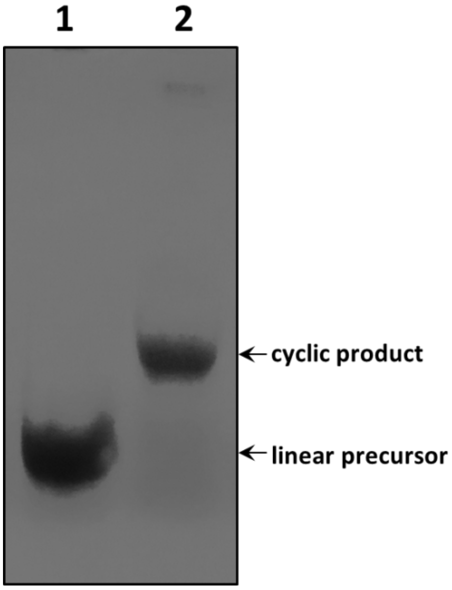

Figure S3. Templated di-imide-mediated cyclisation of linear oligonucleotide (**2**_PA). 13% denaturing PAGE gel analysis of 5′-amine/3′-phosphate oligonucleotide cyclisation to generate circular template with phosphoramidate linkage. Lane 1: linear 5′-amine/3′-phosphate functionalised template; Lane 2: cyclisation reaction mixture.

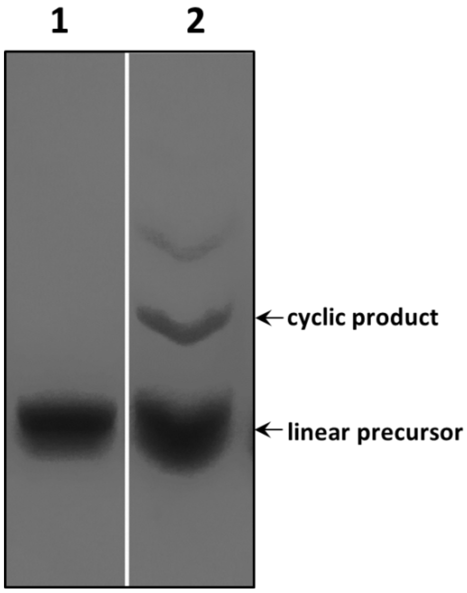

Figure S4. Templated di-imide-mediated cyclisation of template (2_Am). 13% denaturing PAGE gel analysis of 5′-amine/3′-carboxyl oligonucleotide cyclisation to generate circular template with amide linkage. Lane 1: linear 5′-amine/3′-carboxyl functionalised template; Lane 2: cyclisation reaction mixture.

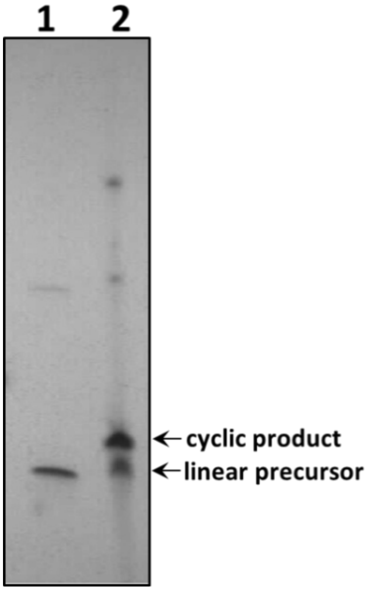

Figure S5. Non-templated click cyclisation of template (**2**_Tz). 8% denaturing PAGE gel analysis of 5′-azide/3′-propargyl oligonucleotides cyclisation to generate circular template with triazole linkage. Lane 1: linear 5′-azide/3′-propargyl functionalised template; Lane 2: cyclisation reaction mixture.

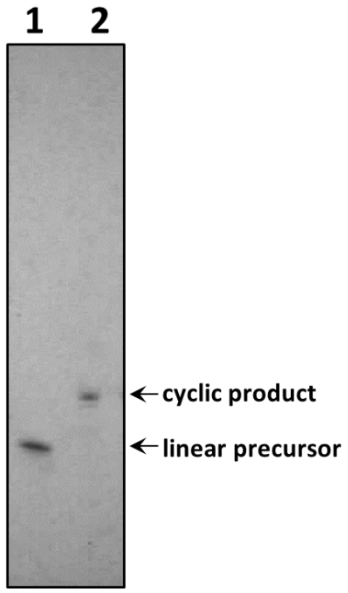

Figure S6. Splint-mediated enzymatic cyclisation of template (**3**_PO_4) using T4 DNA ligase. 12% denaturing PAGE gel analysis of 5′-phosphate/3′-hydroxyl oligonucleotide cyclisation to generate circular template with phosphodiester linkage. Lane 1: linear 5′-phosphate/3′-hydroxyl functionalised template; Lane 2: cyclisation reaction mixture.

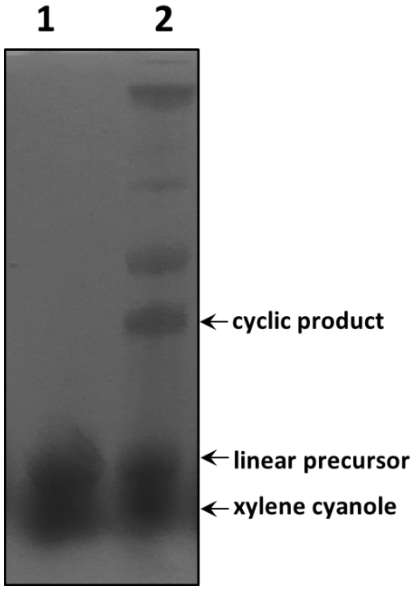

Figure S7. Templated di-imide-mediated cyclisation of template (**3**_PA). 15% denaturing PAGE gel analysis of 5′-amine/3′-phosphate oligonucleotide cyclisation to generate circular template with phosphoramidate linkage. Lane 1: linear 5′-amine/3′-phosphate functionalised template; Lane 2: cyclisation reaction mixture.

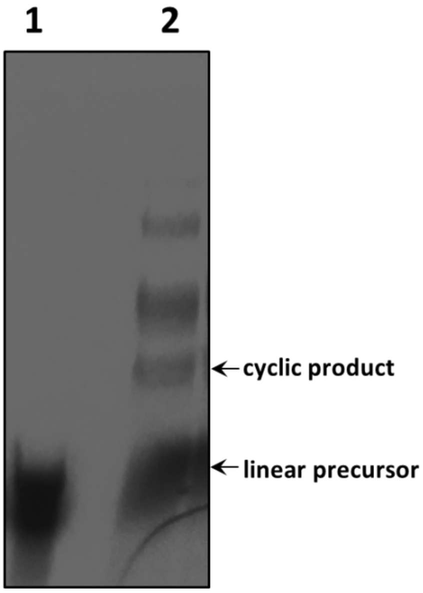

Figure S8. Templated di-imide-mediated cyclisation of template (**3**_Am). 15% denaturing PAGE gel analysis of 5′-amine/3′-carboxyl oligonucleotide cyclisation to generate circular template with amide linkage. Lane 1: linear 5′-amine/3′-carboxyl functionalised template; Lane 2: cyclisation reaction mixture.

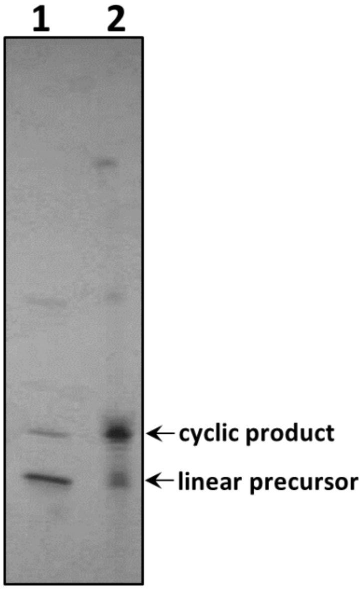

Figure S9. Non-templated click cyclisation of template (**3**_Tz). 12% denaturing PAGE gel analysis of 5′-azide/3′-propargyl oligonucleotide cyclisation to generate circular template with triazole linkage. Lane 1: linear 5′-azide/3′-propargyl functionalised template; Lane 2: cyclisation reaction mixture.

Table S2: Oligonucleotide mass data

Oligonucleotide	Calc. Mass	Found Mass
ODN 1	15473	15474
ODN 2	15457	15458
ODN 3	15451	15450
ODN 4	15474	15474
ODN 5	18964	18966
ODN 6	18948	18949
ODN 7	18942	18942
ODN 8	18965	18967
ODN 9	15012	15014
ODN 10	14997	14998
ODN 11	14976	14975
ODN 12	15014	15013
ODN 13	23180	23182
ODN 14	23180	23182
ODN 15	5539	5540
ODN 16	4939	4941
ODN 17	5010	5012
ODN 18	6420	6421
ODN 19	5507	5507
ODN 20	5540	5540
ODN 21	5643	5643
ODN 22	5557	5556
ODN 23	5670	5670
ODN 24	6030	6030
ODN 25	6030	6031
Cyclic ODN 1 (**1**_PO_4)	15456	15458
Cyclic ODN 2 (**1**_Tz)	15457	15458
Cyclic ODN 3 (**1**_Am)	15433	15434
Cyclic ODN 4 (**1**_PA)	15457	15455
Cyclic ODN 5 (**2**_PO_4)	18947	18949
Cyclic ODN 6 (**2**_Tz)	18948	18950
Cyclic ODN 7 (**2**_Am)	18924	18924
Cyclic ODN 8 (**2**_PA)	18948	18949
Cyclic ODN 9 (**3**_PO_4)	14995	14996
Cyclic ODN 10 (**3**_Tz)	14997	14998
Cyclic ODN 11 (**3**_Am)	14958	14959
Cyclic ODN 12 (**3**_PA)	14997	14997
Cyclic ODN 13 (**4**_PO_4)	23163	23163
Cyclic ODN 14 (**4**_Tz)	23180	23180

UPLC-mass spectrometry characterisation of the linear and circular oligonucleotides was obtained using a Bruker micrOTOF^{TM} II focus ESI-TOF mass spectrometer with an Acquity UPLC system or a Waters Xevo G2-XS QTof mass spectrometer with an Acquity UPLC system. Data were analysed using MassLynx software or Waters UNIFI Scientific Information System software.

UPLC traces and mass spectra of the cyclic DNA templates used in this study

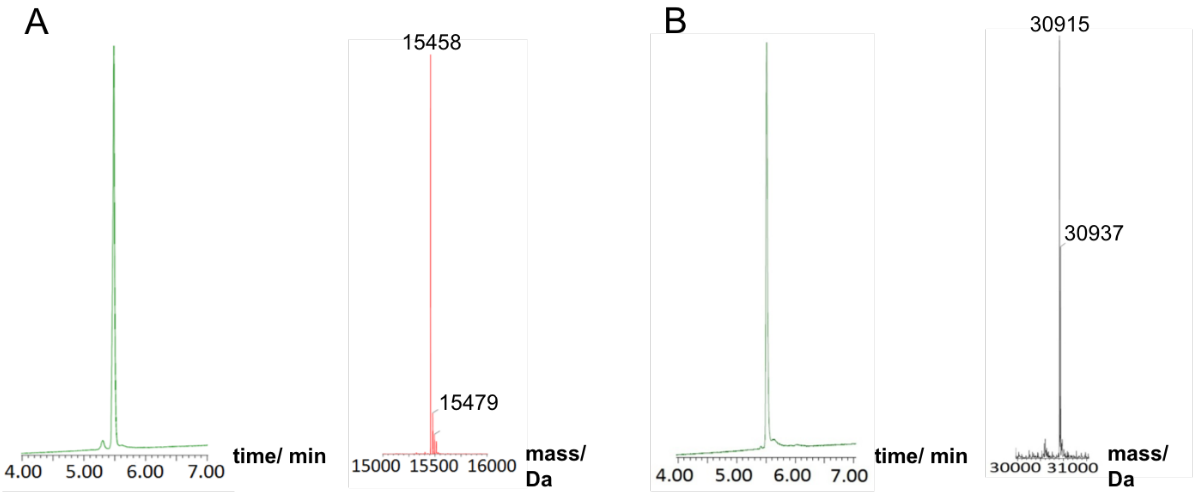

Figure S10. A) Reverse-phase UPLC (UV abs at 260 nm) and mass spectrum (ES$^-$) of the cyclic template **1**_PO$_4$, required 15456 Da, found 15458 Da. The peaks show the product and its Na$^+$ adduct. B) Reverse-phase UPLC (UV abs at 260 nm) and mass spectrum (ES$^-$) of the major, slow migrating side-product extracted from the gel during PAGE purification of **1**_PO$_4$. The side-product is assigned as a cyclic dimer of **1**_PO$_4$: required 30912 Da, found 30915 Da. The peaks show the product and its Na$^+$ adduct.

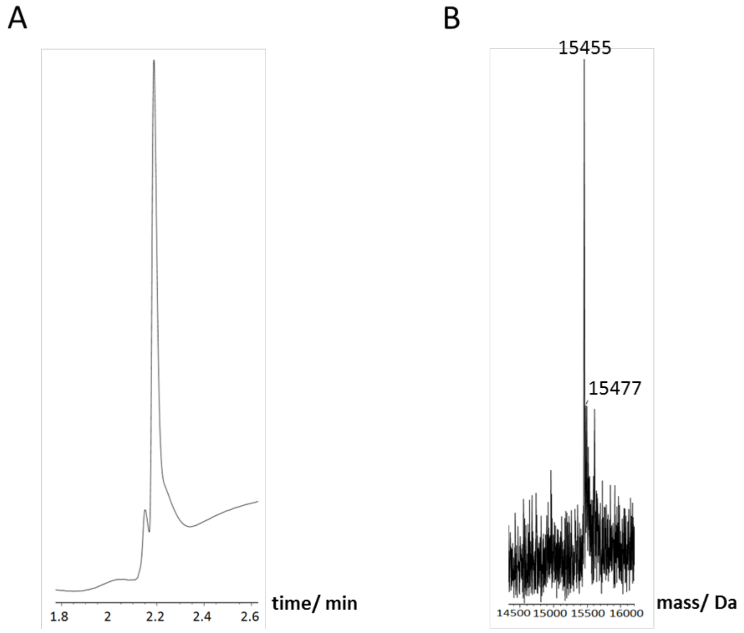

Figure S11. Reverse-phase UPLC (UV abs at 260 nm) and mass spectrum (ES$^-$) of cyclic template **1**_PA, required 15457 Da, found 15455 Da. The peaks show the product and the Na$^+$ adduct.

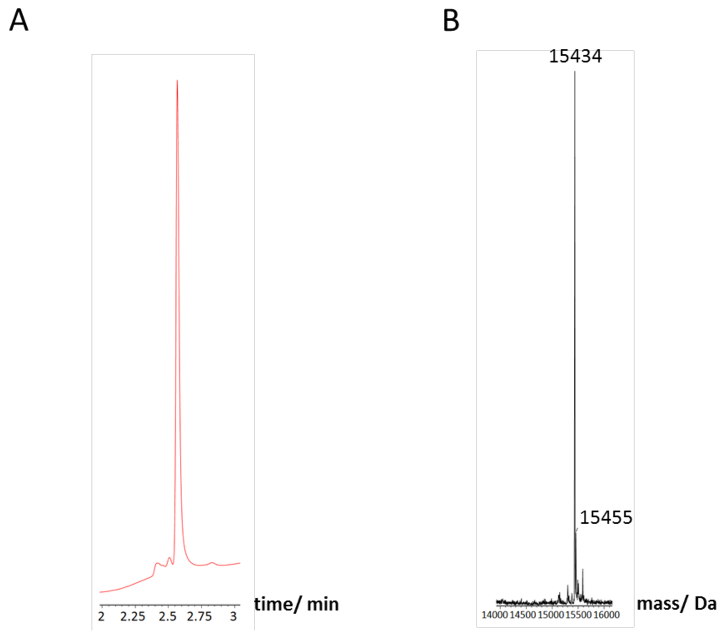

Figure S12. Reverse-phase UPLC (UV abs at 260 nm) and mass spectrum (ES$^-$) of cyclic template **1**_Am, required 15433 Da, found 15434 Da. The peaks show the product and the Na$^+$ adduct.

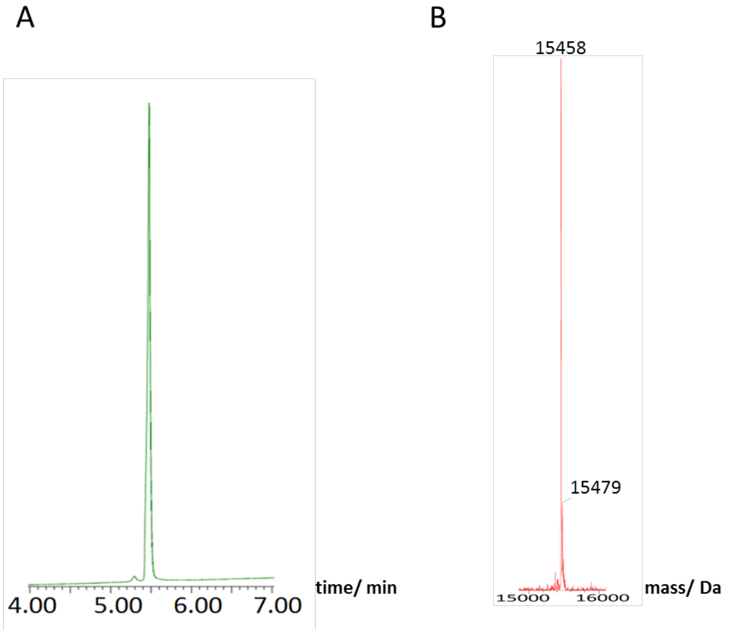

Figure S13. Reverse-phase UPLC (UV abs at 260 nm) and mass spectrum (ES$^-$) of cyclic template **1**_Tz, required 15457 Da, found 15458 Da. The peaks show the product and the Na$^+$ adduct.

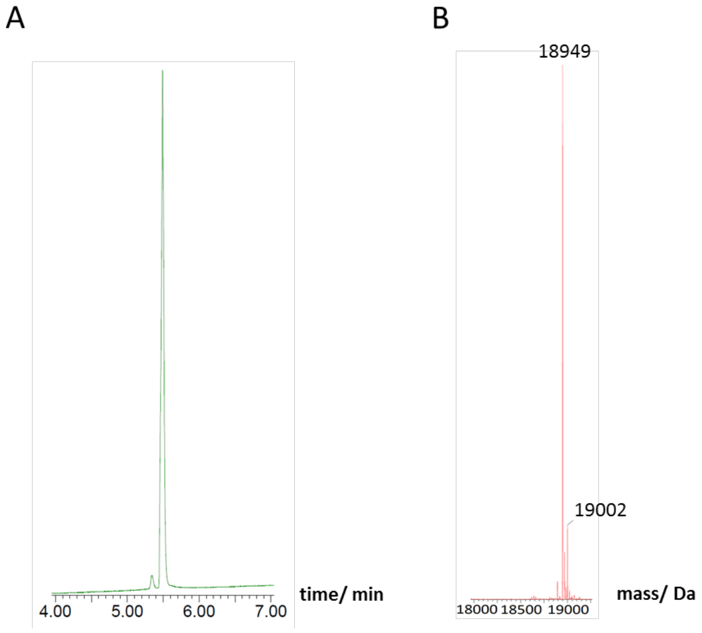

Figure S14. Reverse-phase UPLC (UV abs at 260 nm) and mass spectrum (ES^-) of cyclic template **2**_PO_4, required 18947 Da, found 18949 Da. The peaks show the product and the acrylonitrile adduct.

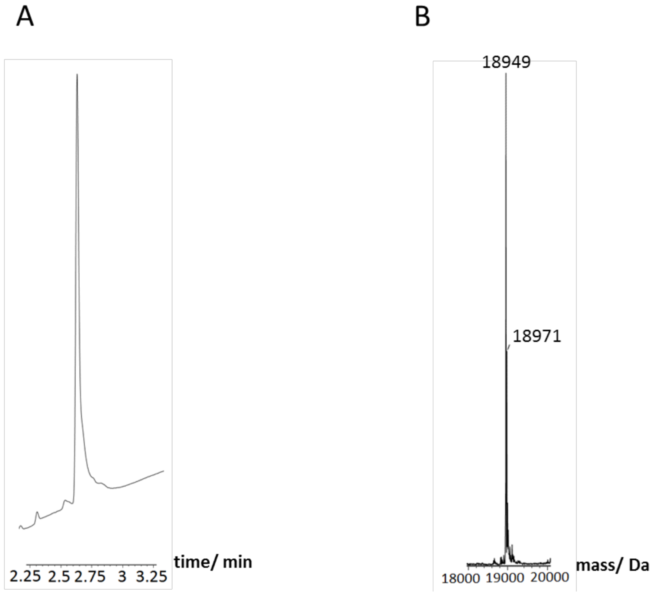

Figure S15. Reverse-phase UPLC (UV abs at 260 nm) and mass spectrum (ES^-) of cyclic template **2**_PA, required 18948 Da, found 18949 Da. The peaks show the product and the Na^+ adduct.

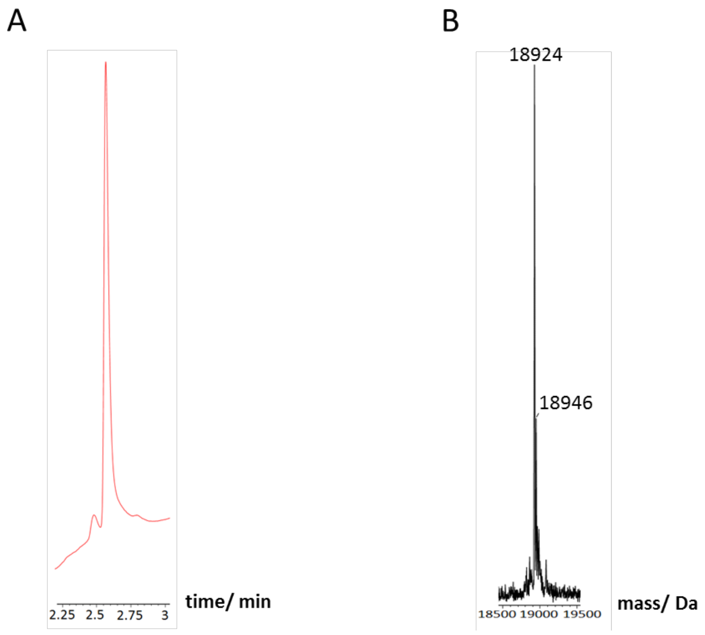

Figure S16. Reverse-phase UPLC (UV abs at 260 nm) and mass spectrum (ES^-) of cyclic template **2**_Am, required 18924 Da, found 18924 Da. The peaks show the product and the Na^+ adduct.

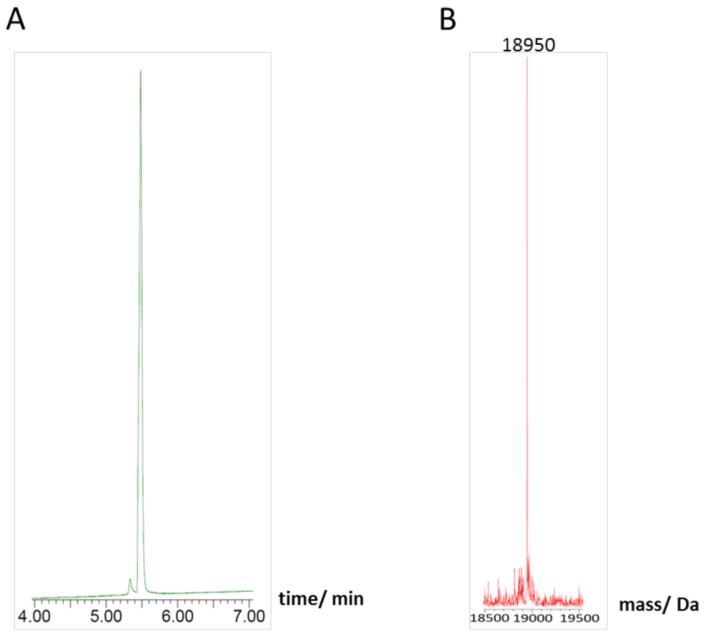

Figure S17. Reverse-phase UPLC (UV abs at 260 nm) and mass spectrum (ES^-) of cyclic template **2**_Tz, required 18948 Da, found 18950 Da.

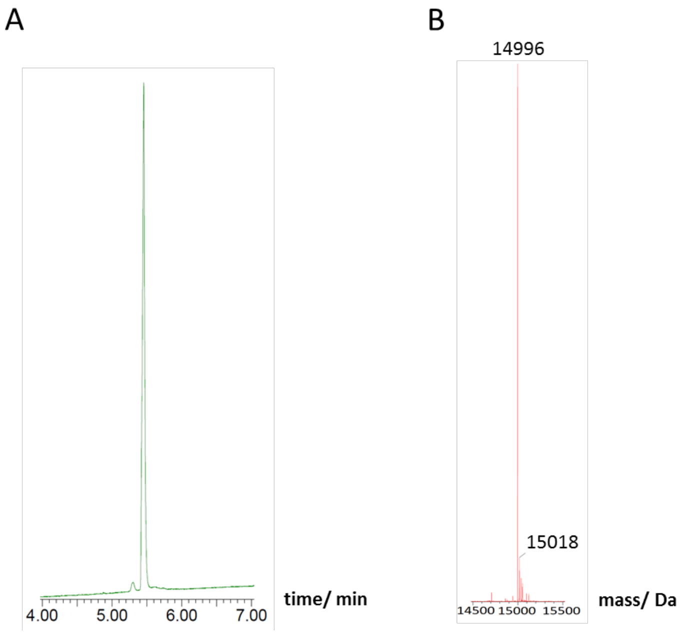

Figure S18. Reverse-phase UPLC (UV abs at 260 nm) and mass spectrum (ES^{-}) of cyclic template **3**_PO_4, required 14995 Da, found 14996 Da. The peaks show the product and the Na^{+} adduct.

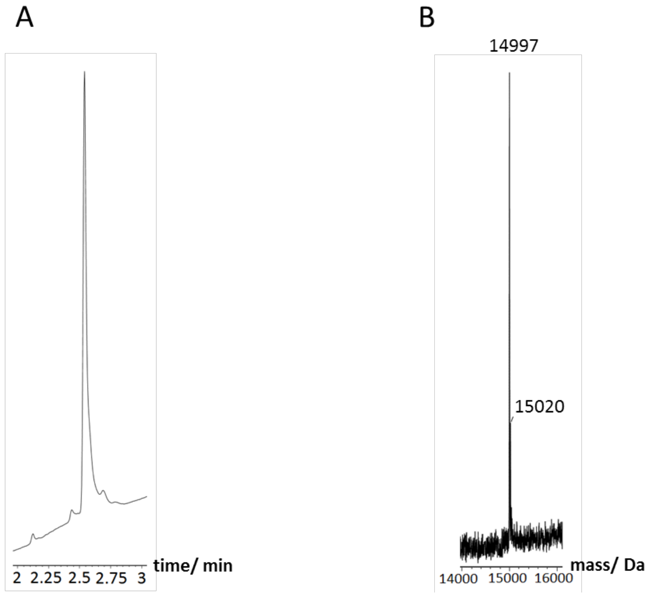

Figure S19. Reverse-phase UPLC (UV abs at 260 nm) and mass spectrum (ES^{-}) of cyclic template **3**_PA, required 14997 Da, found 14997 Da. The peaks show the product and the Na^{+} adduct.

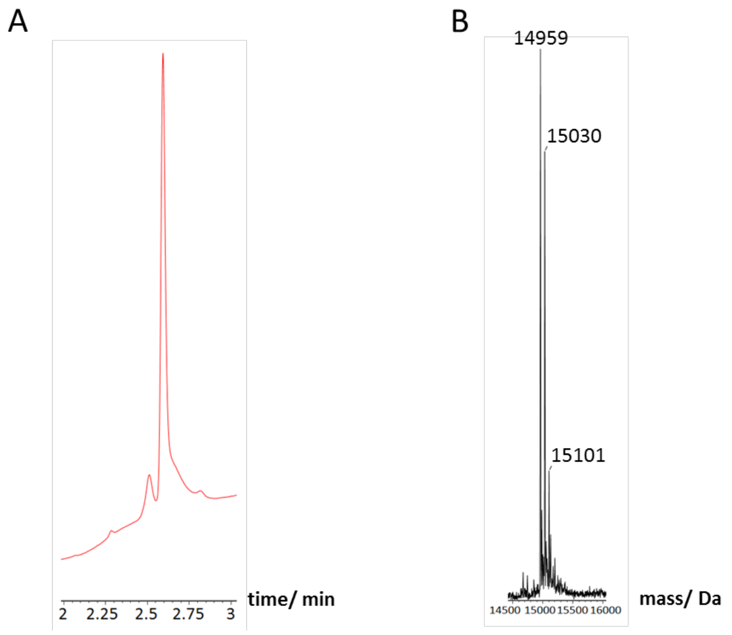

Figure S20. Reverse-phase UPLC (UV abs at 260 nm) and mass spectrum (ES$^-$) of cyclic template **3**_Am, required 14958 Da, found 14959 Da. The identities of the species with molecular weights of 15030 and 15101 are uncertain. They are tentatively assigned as either partially hydrolysed EDC adducts of the cyclic product, or incompletely deprotected analogues containing residual *N*-isobutyryl-functionalised guanine groups.

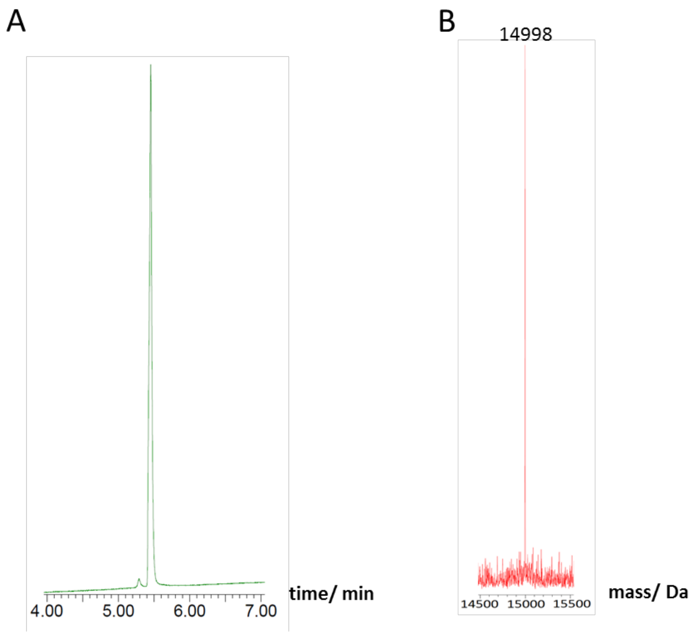

Figure S21. Reverse-phase UPLC (UV abs at 260 nm) and mass spectrum (ES$^-$) of cyclic template **3**_Tz, required 14997 Da, found 14998 Da.

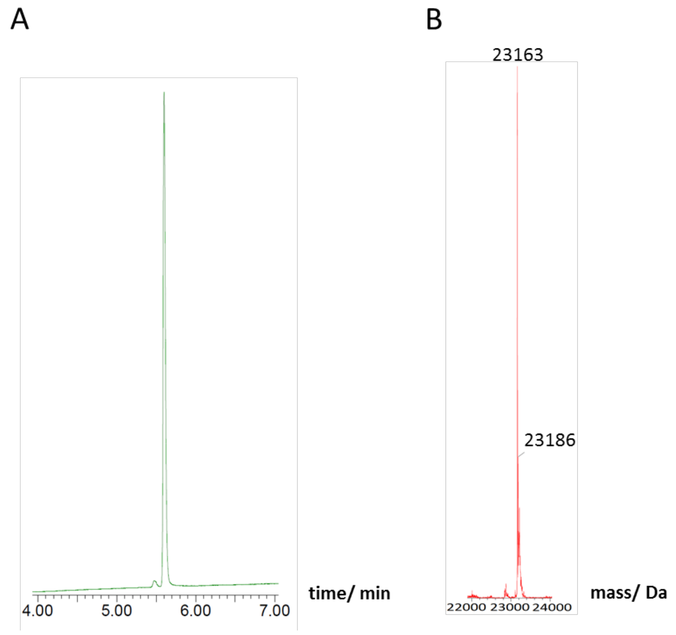

Figure S22. Reverse-phase UPLC (UV abs at 260 nm) and mass spectrum (ES$^-$) of cyclic template **4**_PO$_4$, required 23163 Da, found 23163 Da. The peaks show the product and the Na$^+$ adduct.

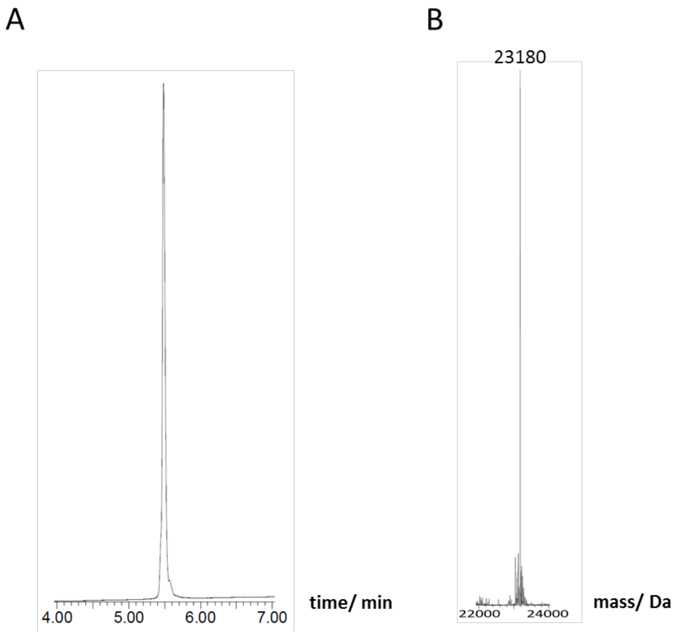

Figure S23. Reverse-phase UPLC (UV abs at 260 nm) and mass spectrum (ES$^-$) of cyclic template **4**_Tz, required 23180 Da, found 23180 Da.

Rolling circle amplification

General procedure for agarose gel electrophoresis

The RCA products were analysed using a 0.8% (w/v) agarose gel with the addition of 0.5X SYBR™ Gold (ThermoFisher). 7.5 µL of sample was mixed with 2.5 µL of 5X GoTaq® Green buffer (Promega) before loading onto the gel. The gel was run at RT (126 V) in 1X TBE buffer and imaged using a G:Box (Syngene).

General procedures for RCA reactions

Comparing the performance of the different templates and backbones in RCA catalysed using Φ-29 DNA polymerase. The following setup was used for each template described: The cyclic template (3 pmol) and primer (12 pmol) were dissolved in 11.2 µL of MilliQ water and 2 µL of $MgSO_4$ (100 mM) and 2 µL of 10X Φ-29 DNA polymerase buffer (500 mM Tris-HCl, pH 7.5 @ 25°C, 100 mM $(NH_4)_2SO_4$, 40 mM DTT, 100 mM $MgCl_2$) were added. The samples were annealed by heating the mixture to 95 °C for 5 min followed by cooling to RT at a rate of 0.5 °C/min. Φ-29 DNA polymerase (10,000 U/ mL, 0.8 µL) and dNTPs (10 mM, 4 µL) were added to the above mixture. The samples were incubated at 30 °C for the time described in the manuscript. The enzyme was then inactivated by heating the samples at 65 °C for 10 min before cooling to 4 °C. The RCA reaction mixture was diluted 5 times with MilliQ water and analysed by 0.8% agarose gel electrophoresis before centrifugation.

Quantification of the RCA rate with Φ-29 DNA polymerase using fluorescent probes

RCA reactions were performed as described above for different lengths of time (0 h, 2 h, 4 h, 6 h, 8 h, 10 h, or 20 h) using **1**_PO_4, **1**_PA, **1**_Am and **1**_Tz and their corresponding primers. After heat inactivation of the enzyme, the samples were diluted 5 times with 10 mM EDTA in MilliQ water. 10 µL of each sample was mixed with a 10 µL solution composed of fluorescent probe (100 µM) and NaCl (1 M) and incubated at RT for 2 h. 9 µL of each sample was mixed with 3 µL of 5X GoTaq® Green buffer (Promega) and analysed by 0.8% agarose gel electrophoresis (cast without SYBR Gold). The gels were run at RT (100 V) and imaged using a G:Box gel documentation system (Syngene) with excitation at 520 nm under the Cy3 fluorescence channel. Afterwards, the gels were stained with SYBR Gold (1X) at RT for 15 min, and re-imaged with excitation at 302 nm.

RCA reaction catalysed using Φ-29 DNA polymerase with 10 mM Mg^{2+} and single stranded binding protein T4 gene 32 for cyclic templates (1_PO_4 and 1_PA)

The cyclic template (4.5 pmol) and primer (18 pmol) were dissolved in 19.5 µL MilliQ water and 3 µL of 10X Φ-29 DNA polymerase buffer was added. The solution was annealed by heating the mixture to 95 °C for 5 min followed by cooling to RT at a rate of 0.5 °C/min. Φ-29 DNA polymerase (10,000 U/ mL, 1.2 µL), dNTPs (10 mM, 6 µL) and T4 gene 32 protein (10 mg/ mL, 0.3 µL) were added to the above mixture (The concentration of Mg^{2+} and T4 gene 32 protein were chosen based on previous literature (4)). The RCA reactions were incubated at 30 °C for 20 h. The enzyme was then inactivated by heating

the samples at 65 °C for 10 min before cooling to 4 °C. The original RCA reaction mixture was diluted to 100 µL with MilliQ water and then analysed by 0.8% agarose gel electrophoresis.

Measurement of fluorescence intensity of the RCA products stained with SYBR Green I, SYBR Green II and SYBR Gold

RCA reactions were performed as described above. After heat inactivation of the polymerase enzyme the samples were diluted 500 times with a solution of 20 mM of EDTA in MilliQ water.100 µL of buffer (1X TBE, 0.1% triton X-100, 1X SYBR Green I, 1X SYBR Green II or 1X SYBR Gold) was added into a 96-well plate followed by adding 5 µL of diluted RCA product. Before each measurement the 96-well plate was shaken three times 10 minutes apart (1 min, 300 RPM). The fluorescence intensity of the solution was then measured using a CLARIOstar microplate reader (BMG LABTECH, Ortenberg, Germany). Three independent readings were taken for each RCA sample. For SYBR Green I measurements the parameters used were Excitation: 492-8; Dichroic filter: 514.2; Emission: 526-8. For SYBR Green II measurements the parameters used were Excitation: 492-8; Dichroic filter: 514.2; Emission: 526-8. For SYBR Gold measurements the parameters used were Excitation: 495-8; Dichroic filter: 514.2; Emission: 537-15.

RCA reaction catalysed using Bst 2.0 DNA polymerase with 20 mM Mg^{2+}

The cyclic template (3 pmol) and primer (12 pmol) were dissolved in 10 µL MilliQ water and 3.6 µL of 100 mM $MgSO_4$ and 2 µL of 10X isothermal amplification buffer (200 mM Tris-HCl, pH 8.8 @ 25°C, 100 mM $(NH4)_2SO_4$, 500 mM KCl, 20 mM MgSO4, 0.1% Tween® 20) were added. The sample was annealed by heating the mixture to 95 °C for 5 min followed by cooling to RT at a rate of 0.5 °C/min. Bst 2.0 DNA polymerase (8000 U/ mL, 0.4 µL) and dNTPs (10 mM, 4 µL) were then added. The RCA reactions were carried out at 61.5 °C for 20 h. The enzyme was then inactivated by heating the samples at 80 °C for 20 min before cooling to 4 °C. The original RCA reaction mixture was diluted 5 times by MilliQ water and then analysed by 0.8% agarose gel electrophoresis.

DNA nanoflower experiments

Using the cyclic templates to prepare DNA-nanoflowers (DNA-NFs)

A solution of cyclic template (3 pmol), primer (12 pmol), 2 µL $MgSO_4$ (100 mM aqueous solution) and 2 µL of 10X Φ-29 DNA polymerase buffer (500 mM Tris-HCl, pH 7.5 @ 25°C, 100 mM $(NH_4)_2SO_4$, 40 mM DTT, 100 mM $MgCl_2$) in MilliQ water with a final volume of 15.2 µL was first annealed by heating the mixture to 95 °C for 5 min followed by cooling to RT at a rate of 0.5 °C/min. Φ-29 DNA polymerase (10,000 U/ mL, 0.8 µL) and dNTPs (10 mM, 4 µL) were added to the above mixture and briefly vortexed. The samples were incubated at 30 °C for either 8 h or 20 h using a BioRad T100 thermocycler. The enzyme was then inactivated by heating the samples at 65 °C for 10 min before cooling to 4 °C. 80 µL of MilliQ water was added and the samples were centrifuged. The resultant precipitate was collected by centrifugation and washed 5 times with MilliQ water followed by resuspending in 20 µL of MilliQ water. 10 µL of each of the RCA precipitate suspensions were loaded onto silicon wafer chips and dried

at 55 °C for 15 min. The samples were then coated with gold before imaging using a Zeiss Sigma 300 Field Emission Gun Scanning Electron Microscope. The resuspended precipitate solution was also analysed by agarose gel electrophoresis.

Comparing the effect of Mg^{2+} concentration on DNA loading of DNA-NFs

A solution of cyclic template (3 pmol), primer (12 pmol), $MgSO_4$ (either 0 µL, 0.5 µL, 1 µL, 2 µL or 3 µL of a 100 mM aqueous solution) and 2 µL of 10X Φ-29 DNA polymerase buffer (500 mM Tris-HCl, pH 7.5 @ 25°C, 100 mM $(NH_4)_2SO_4$, 40 mM DTT, 100 mM $MgCl_2$) in MilliQ water with a final volume of 15.2 µL was first annealed by heating the mixture to 95 °C for 5 min followed by cooling to RT at a rate of 0.5 °C/min. Φ-29 DNA polymerase (10,000 U/ mL, 0.8 µL) and dNTPs (10 mM, 4 µL) were added to the above mixture and briefly vortexed. The samples were incubated at 30 °C for 20 h using a BioRad T100 thermocycler. The enzyme was then inactivated by heating the samples at 65 °C for 10 min before cooling to 4 °C. The samples were centrifuged and the supernatant was carefully collected. The resultant precipitate was collected by centrifugation and washed 5 times with MilliQ water followed by resuspending in 20 µL of MilliQ water. 7.5 µL of the supernatant or the precipitate suspension were then analysed by 0.8% agarose gel electrophoresis.

Comparing the effect of Mg^{2+} concentration on DNA-NF size

RCA was performed as described in the 'comparing the effect of Mg^{2+} concentration on DNA loading of DNA-NFs' section above using cyclic templates **1**_PO_4 and **1**_PA. 10 µL of each of the RCA precipitate suspensions were loaded onto silicon wafer chips and dried at 55 °C for 15 min. The samples were then coated with gold before imaging using a Zeiss Sigma 300 Field Emission Gun Scanning Electron Microscope.

Images of agarose gels used to analyse the products from the RCA reactions

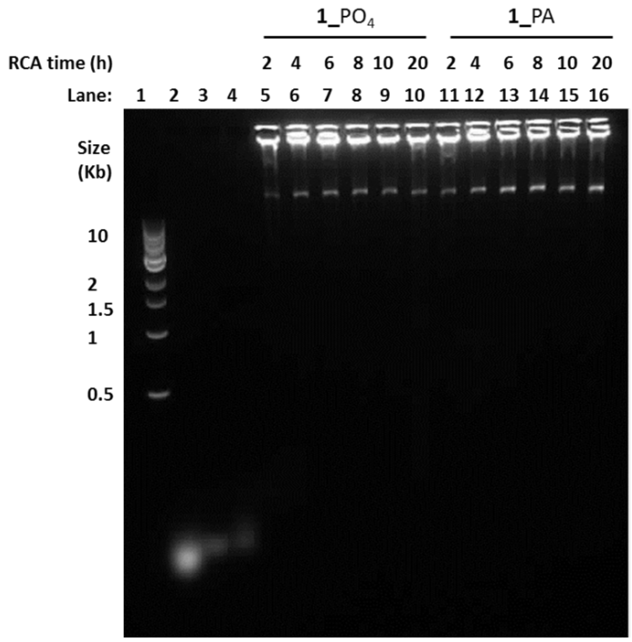

Figure S24. 0.8% agarose gel analysis of original RCA reaction mixture from cyclic **1**_PO_4 and cyclic **1**_PA. The RCA was performed in the presence of 20 mM Mg^{2+} at different time points using Φ-29 DNA polymerase. Lane 1: 1 kb DNA ladder; Lane 2: splint (ODN 15); Lane 3: cyclic unmodified template (**1**_PO_4); Lane 4: cyclic phosphoramidate template (**1**_PA).

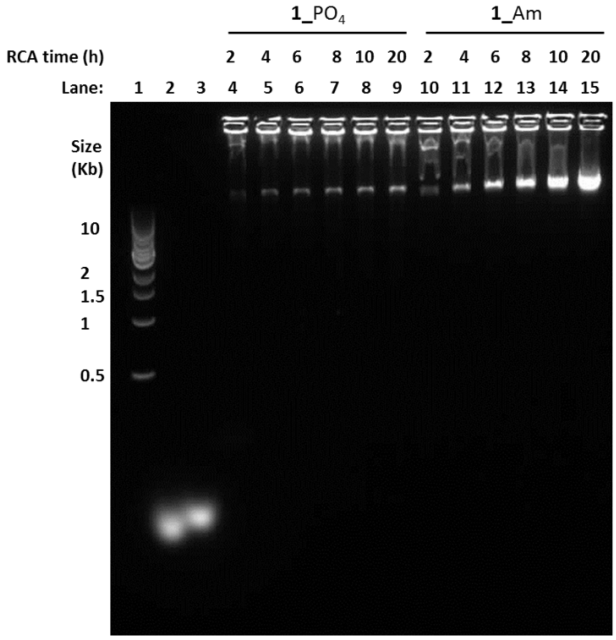

Figure S25. 0.8% agarose gel analysis of original RCA reaction mixture from cyclic **1**_PO_4 and cyclic **1**_Am. The RCA was performed in the presence of 20 mM Mg^{2+} at different time points using Φ-29 DNA polymerase. Lane 1: 1 kb DNA ladder; Lane 2: splint (ODN 15); Lane 3: splint for **1**_Am (ODN 19).

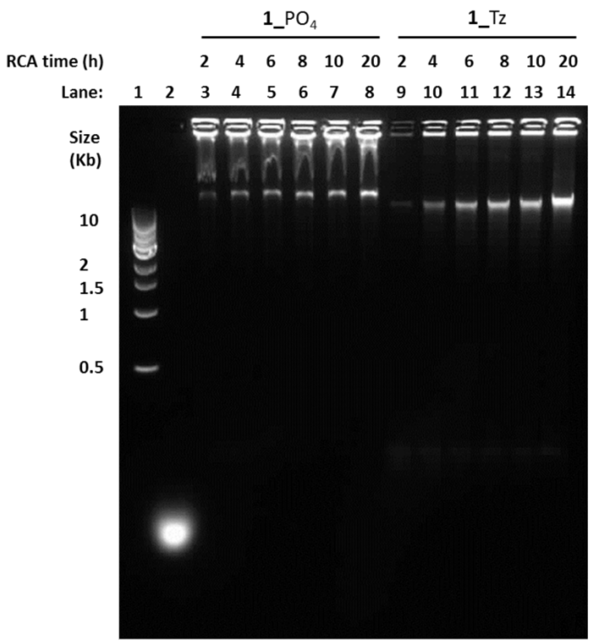

Figure S26. 0.8% agarose gel analysis of original RCA reaction mixture from cyclic **1**_PO_4 and cyclic **1**_Tz. The RCA was performed in the presence of 20 mM Mg^{2+} at different time points using Φ-29 DNA polymerase. Lane 1: 1 kb DNA ladder; Lane 2: splint (ODN 15).

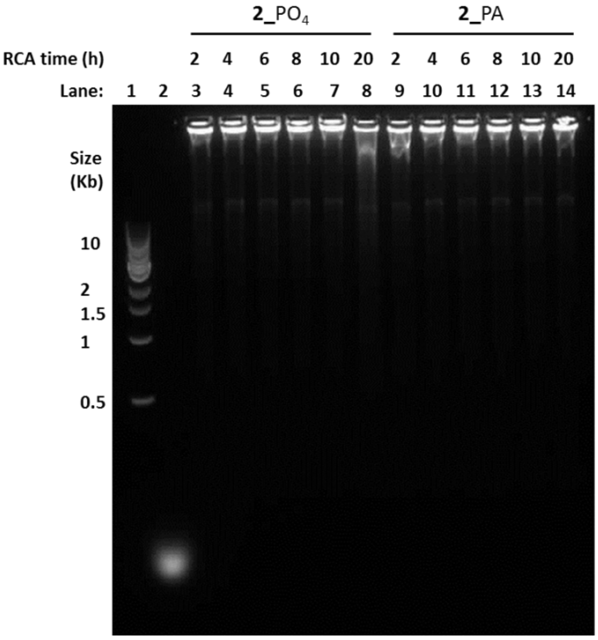

Figure S27. 0.8% agarose gel analysis of original RCA reaction mixture from cyclic **2**_PO_4 and cyclic **2**_PA. The RCA was performed in the presence of 20 mM Mg^{2+} at different time points using Φ-29 DNA polymerase. After finishing the RCA reactions, EDTA (20 mM final concentration) was added to the solution in order to break down the precipitate releasing the extended RCA products. Lane 1: 1 kb DNA ladder; Lane 2: splint (ODN 16).

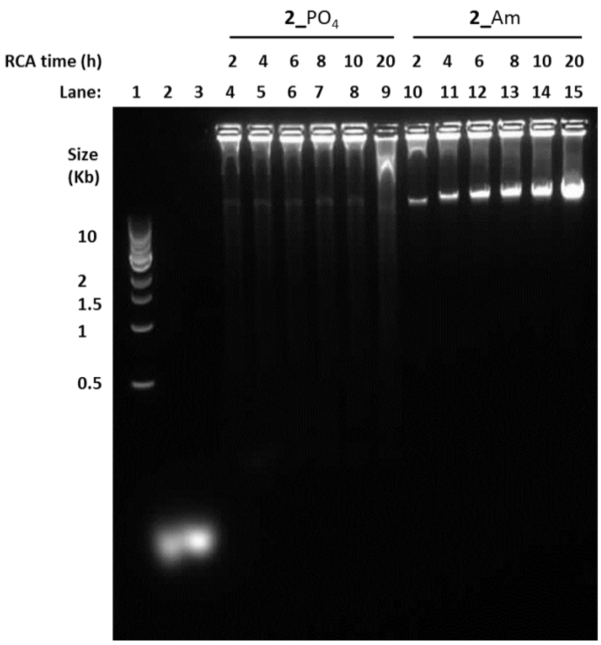

Figure S28. 0.8% agarose gel analysis of original RCA reaction mixture from cyclic **2**_PO_4 and cyclic **2**_Am. The RCA was performed in the presence of 20 mM Mg^{2+} at different time points using Φ-29 DNA polymerase. After finishing the RCA reactions, EDTA (20 mM final concentration) was added to the solution in order to break down the precipitate releasing the extended RCA products. Lane 1: 1 kb DNA ladder; Lane 2: splint (ODN 16); Lane 3: splint for **2**_Am (ODN 20).

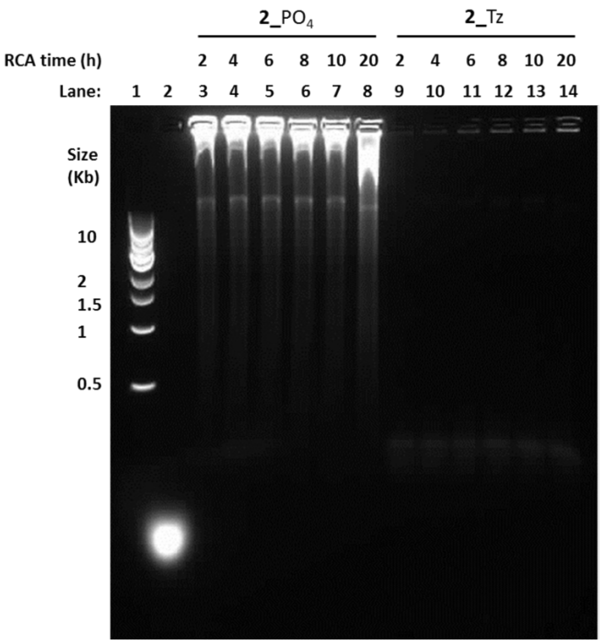

Figure S29. 0.8% agarose gel analysis of original RCA reaction mixture from cyclic **2**_PO_4 and cyclic **2**_Tz. The RCA was performed in the presence of 20 mM Mg^{2+} at different time points using Φ-29 DNA polymerase. After finishing the RCA reactions, EDTA (20 mM final concentration) was added to the solution in order to break down the precipitate releasing the extended RCA products. Lane 1: 1 kb DNA ladder; Lane 2: splint (ODN 16).

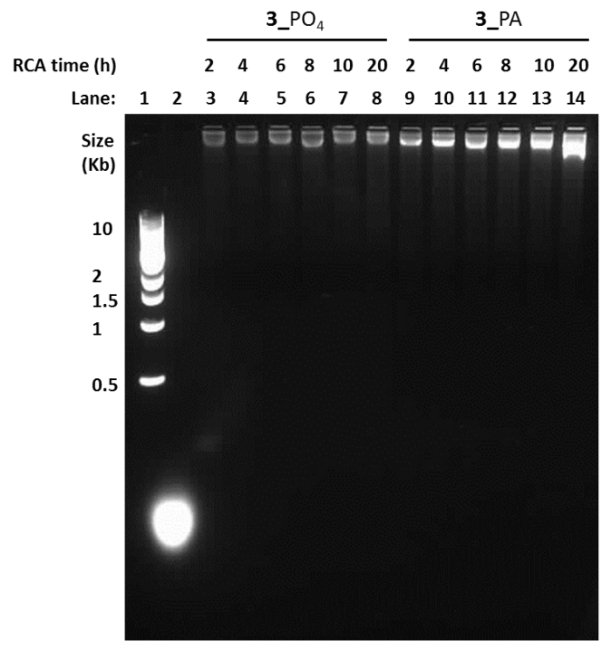

Figure S30. 0.8% agarose gel analysis of RCA supernatant from cyclic **3**_PO_4 and cyclic **3**_PA. The RCA was performed in the presence of 20 mM Mg^{2+} at different time points using Φ-29 DNA polymerase. After finishing the RCA reactions, EDTA (20 mM final concentration) was added to the solution in order to break down the precipitate releasing the extended RCA products. However, the precipitate was still observed. Lane 1: 1 kb DNA ladder; Lane 2: splint (ODN 17).

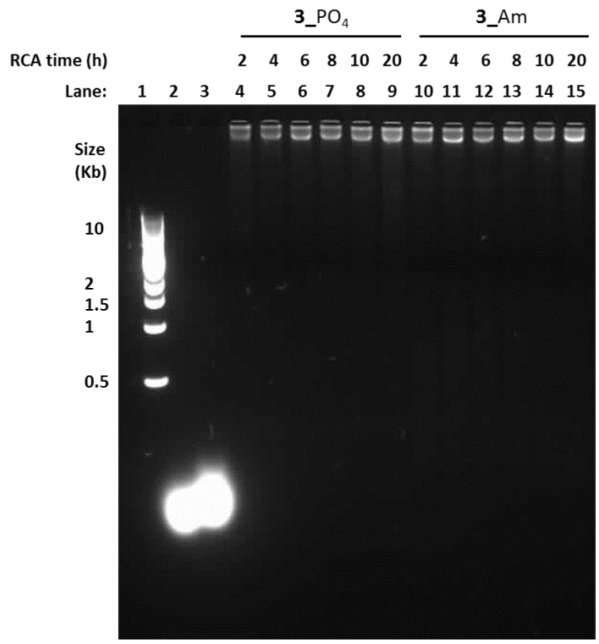

Figure S31. 0.8% agarose gel analysis of RCA supernatant from cyclic **3**_PO_4 and cyclic **3**_Am. The RCA was performed in the presence of 20 mM Mg^{2+} at different time points using Φ-29 DNA polymerase. After finishing the RCA reactions, EDTA (20 mM final concentration) was added to the solution in order to break down the precipitate releasing the extended RCA products. However, the precipitate was still observed. Lane 1: 1 kb DNA ladder; Lane 2: splint (ODN 17); Lane 3: splint for **3**_Am (ODN 21).

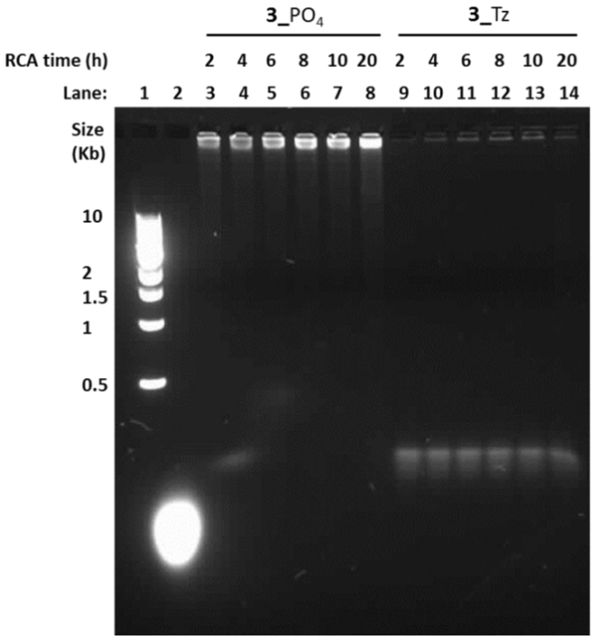

Figure S32. 0.8% agarose gel analysis of RCA supernatant from cyclic **3**_PO_4 and cyclic **3**_Tz. The RCA was performed in the presence of 20 mM Mg^{2+} at different time points using Φ-29 DNA polymerase. After finishing the RCA reactions, EDTA (20 mM final concentration) was added to the solution in order to break down the precipitate releasing the extended RCA products. However, precipitate was still observed. Lane 1: 1 kb DNA ladder; Lane 2: splint (ODN 17).

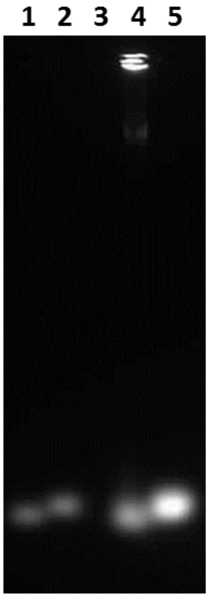

Figure S33. Testing the hybridisation probe assay. 0.8% agarose gel analysis of probing the amplified products from Φ-29-mediated RCA of the cyclic template **1**_PO_4 after incubation with fluorescent probes. lane 1: fluorescent probe ODN 24; lane 2: fluorescent probe ODN 25 (scrambled version of ODN 24); lane 3: RCA products without probes; lane 4: after hybridisation of RCA products with fluorescent probe ODN 24; lane 5: after hybridization of RCA products with scrambled fluorescent probe ODN 25. The gel was imaged using a G:Box (Syngene) with excitation at 520 nm under the Cy3 fluorescence channel.

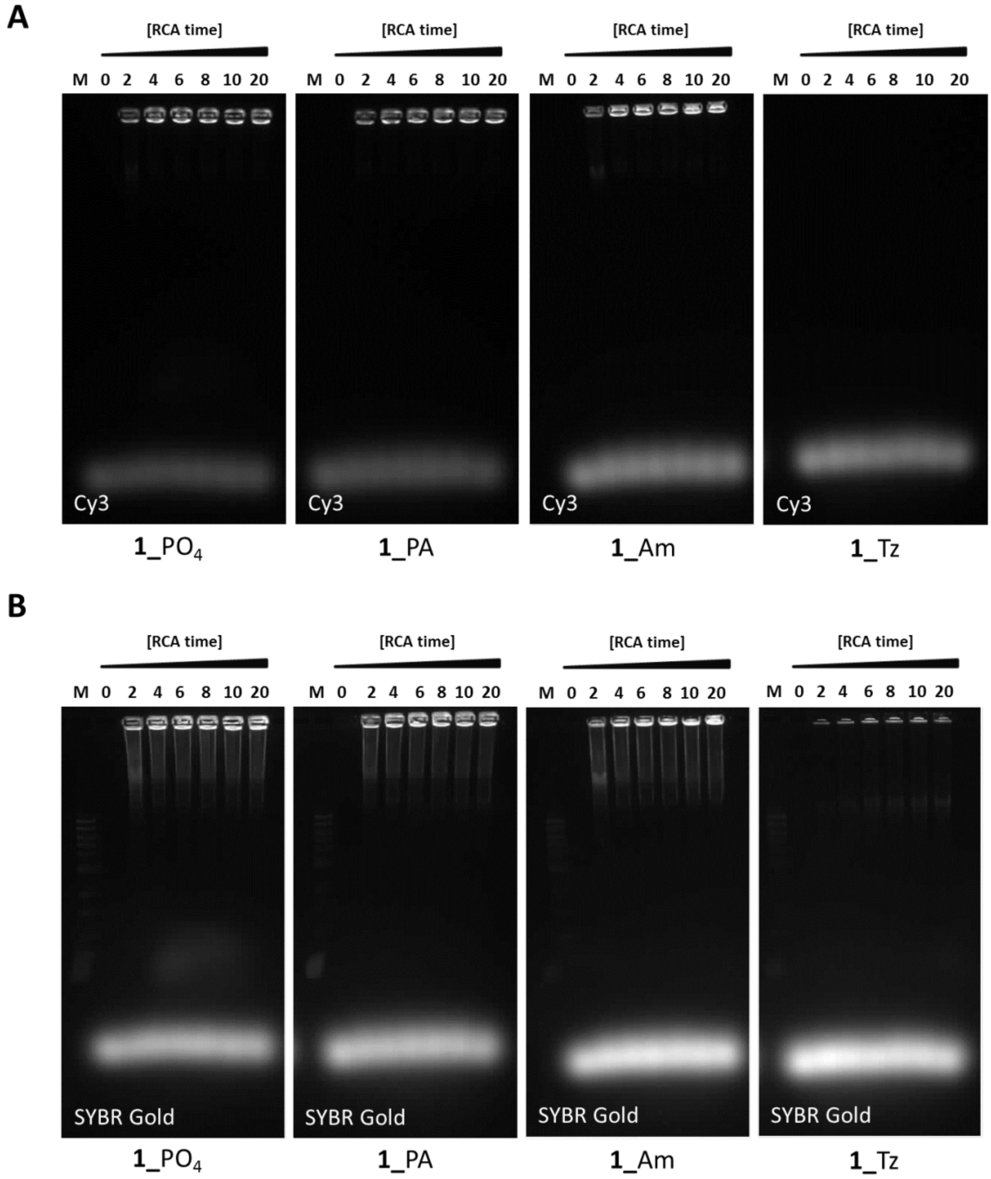

Figure S34. Probing the amplified products from Φ-29-mediated RCA of the cyclic template **1**_PO_4, **1**_PA, **1**_Am and **1**_Tz using fluorescent probe (ODN 24). Aliquots of the RCA reactions were stopped at regular two hour intervals for a total period of 20 hours. A Cy3-labelled fluorescent probe with a sequence which is complementary to the RCA products was incubated with each aliquot and the mixtures were analysed by 0.8% agarose gel electrophoresis. The gels were imaged with excitation at 520 nm under the Cy3 fluorescence channel and re-imaged after SYBR Gold staining with excitation at 302 nm using a G:Box (Syngene).

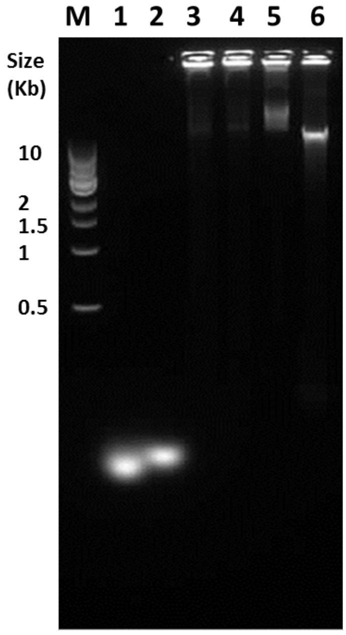

Figure S35. 0.8% agarose gel analysis of precipitate from cyclic **1**_PO_4, cyclic **1**_PA, cyclic **1**_Am and cyclic **1**_Tz. The RCA was performed in the presence of 20 mM Mg^{2+} for 8 h using Φ-29 DNA polymerase. M: 1 kb DNA ladder; Lane 2: splint (ODN 15); Lane 3; splint for cyclic **1**_Am (ODN 19); Lane 3: cyclic **1**_PO_4; Lane 4: cyclic **1**_PA; Lane 5: cyclic **1**_Am; Lane 6: cyclic **1**_Tz.

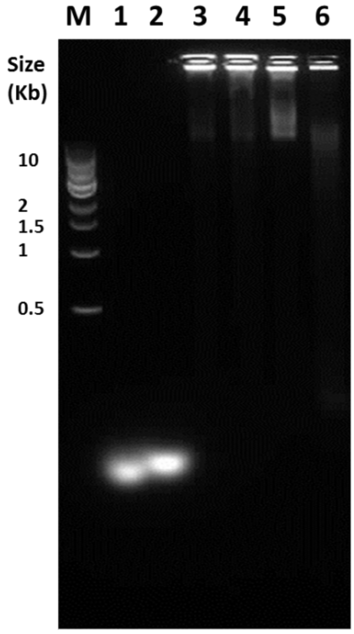

Figure S36. 0.8% agarose gel analysis of precipitate from cyclic **1**_PO_4, cyclic **1**_PA, cyclic **1**_Am and cyclic **1**_Tz. The RCA was performed in the presence of 20 mM Mg^{2+} for 20 h using Φ-29 DNA polymerase. M: 1 kb DNA ladder; Lane 2: splint (ODN 15); Lane 3; splint for cyclic **1**_Am (ODN 19); Lane 3: cyclic **1**_PO_4; Lane 4: cyclic **1**_PA; Lane 5: cyclic **1**_Am; Lane 6: cyclic **1**_Tz.

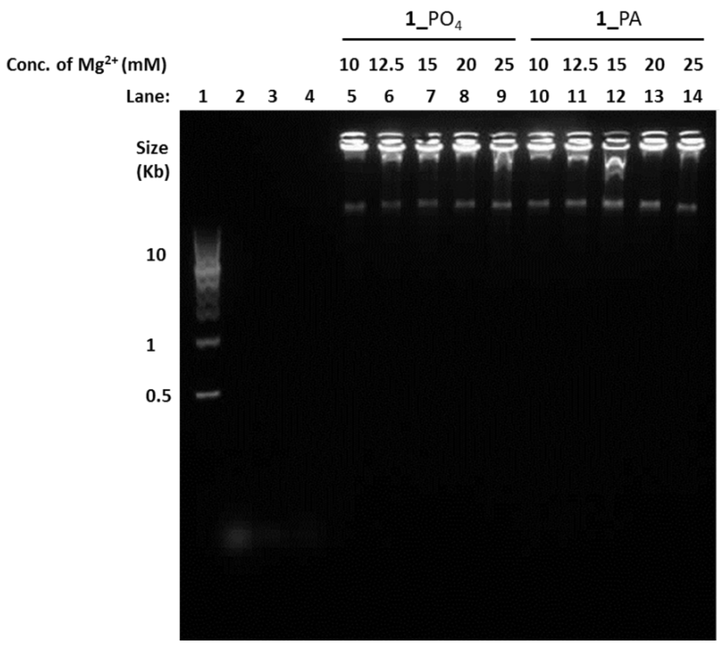

Figure S37. 0.8% agarose gel analysis of supernatant from cyclic **1**_PO$_4$ and cyclic **1**_PA. The RCA was performed for 20 h at different concentrations of Mg^{2+} using Φ-29 DNA polymerase. Lane 1: 1 kb DNA ladder; Lane 2: splint (ODN 15); Lane 3: cyclic unmodified template (**1**_PO$_4$); Lane 4: cyclic phosphoramidate template (**1**_PA).

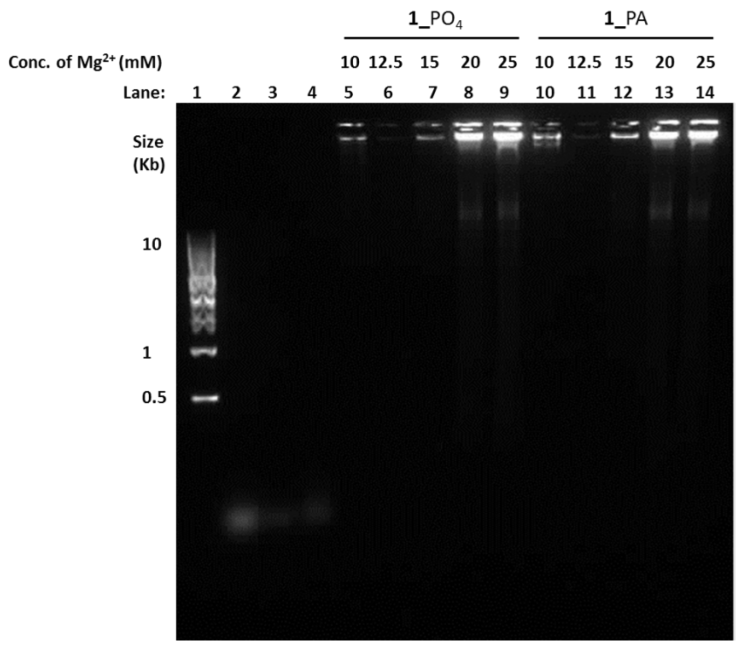

Figure S38. 0.8% agarose gel analysis of precipitate from cyclic **1**_PO$_4$ and cyclic **1**_PA. The RCA was performed for 20 h at different concentrations of Mg^{2+} using Φ-29 DNA polymerase. Lane 1: 1 kb DNA ladder; Lane 2: splint (ODN 15); Lane 3: cyclic unmodified template (**1**_PO$_4$); Lane 4: cyclic phosphoramidate template (**1**_PA).

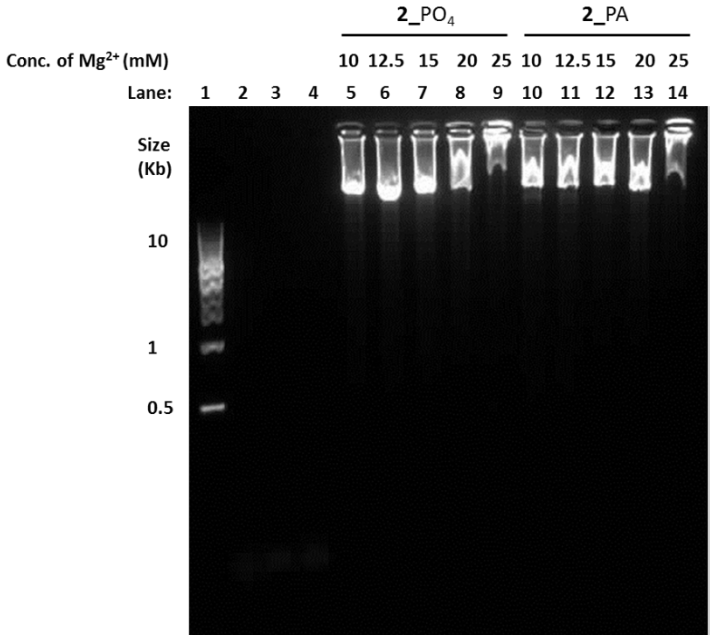

Figure S39. 0.8% agarose gel analysis of supernatant from cyclic **2**_PO_4 and cyclic **2**_PA. The RCA was performed for 20 h at different concentrations of Mg^{2+} using Φ-29 DNA polymerase. Lane 1: 1 kb DNA ladder; Lane 2: splint (ODN 16); Lane 3: cyclic unmodified template (**2**_PO_4); Lane 4: cyclic phosphoramidate template (**2**_PA).

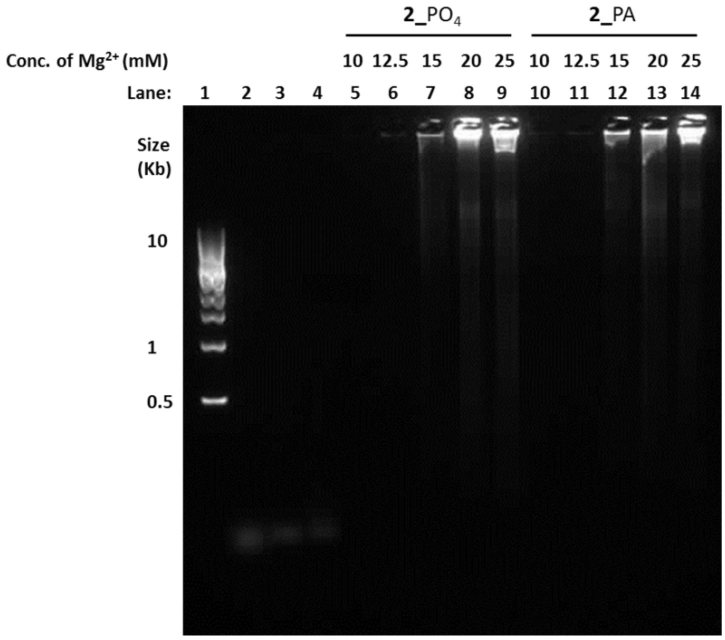

Figure S40. 0.8% agarose gel analysis of precipitate from cyclic **2**_PO_4 and cyclic **2**_PA. The RCA was performed for 20 h at different concentrations of Mg^{2+} using Φ-29 DNA polymerase. Lane 1: 1 kb DNA ladder; Lane 2: splint (ODN 16); Lane 3: cyclic unmodified template (**2**_PO_4); Lane 4: cyclic phosphoramidate template (**2**_PA).

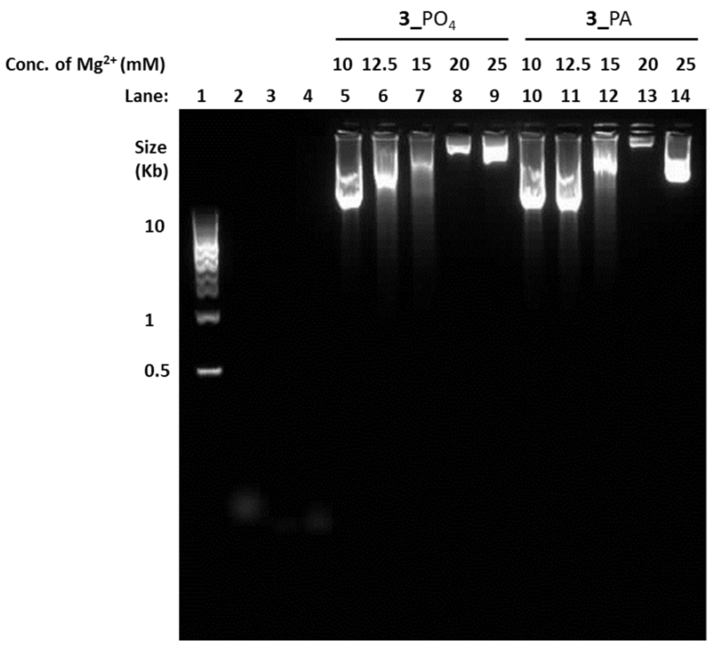

Figure S41. 0.8% agarose gel analysis of supernatant from cyclic **3**_PO4 and cyclic **3**_PA. The RCA was performed for 20 h at different concentrations of Mg^{2+} using Φ-29 DNA polymerase. Lane 1: 1 kb DNA ladder; Lane 2: splint (ODN 17); Lane 3: cyclic unmodified template (**3**_PO4); Lane 4: cyclic phosphoramidate template (**3**_PA).

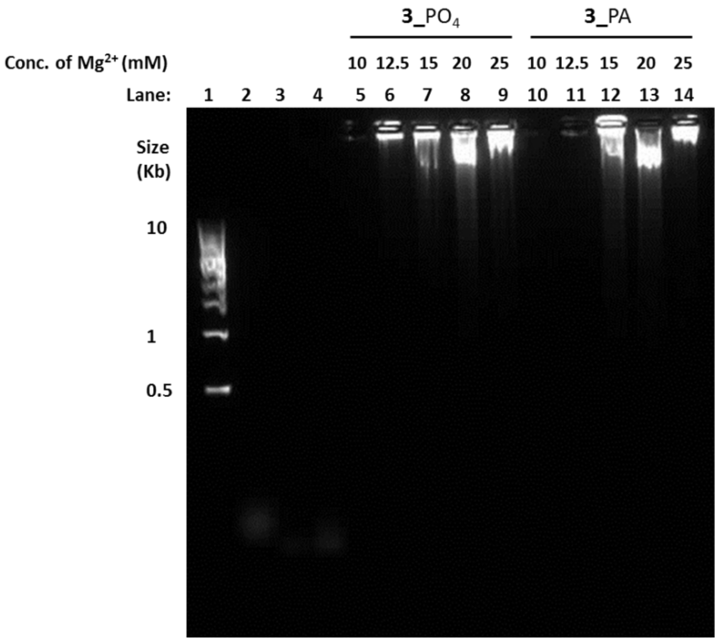

Figure S42. 0.8% agarose gel analysis of precipitate from cyclic **3**_PO4 and cyclic **3**_PA. The RCA was performed for 20 h at different concentrations of Mg^{2+} using Φ-29 DNA polymerase. Lane 1: 1 kb DNA ladder; Lane 2: splint (ODN 17); Lane 3: cyclic unmodified template (**3**_PO4); Lane 4: cyclic phosphoramidate template (**3**_PA).

SEM images used to measure the particle size

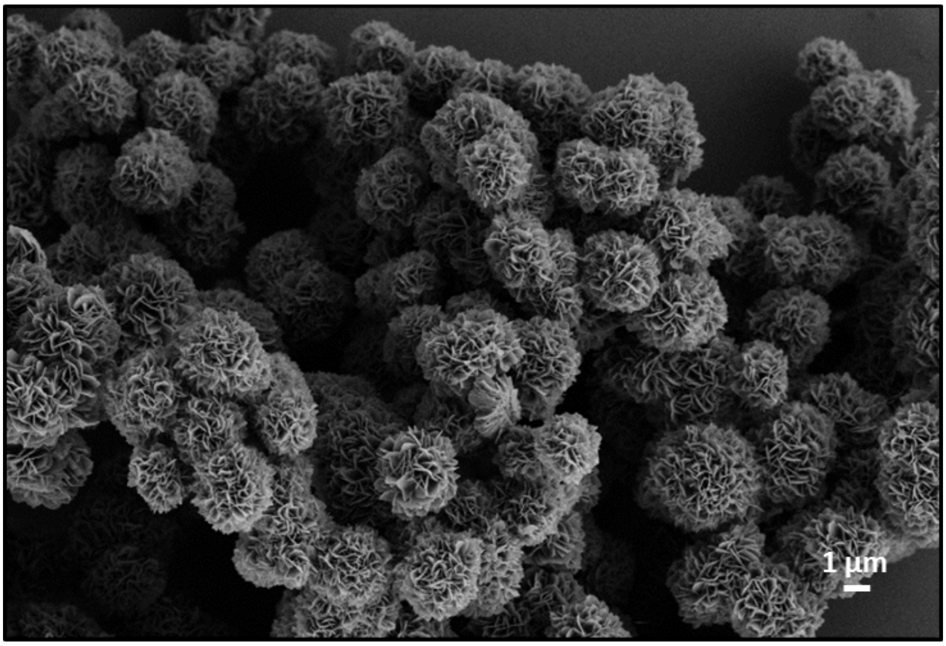

Figure S43. SEM image used to measure the size distribution of the particles generated from cyclic **1**_PO_4. The RCA was carried out for 20 h in the presence of 10 mM Mg^{2+} using Φ-29 DNA polymerase. ImageJ was used to measure the average diameter of particles: 2.98 ± 0.22 μm, n = 20 (n refers to the number of measured particles).

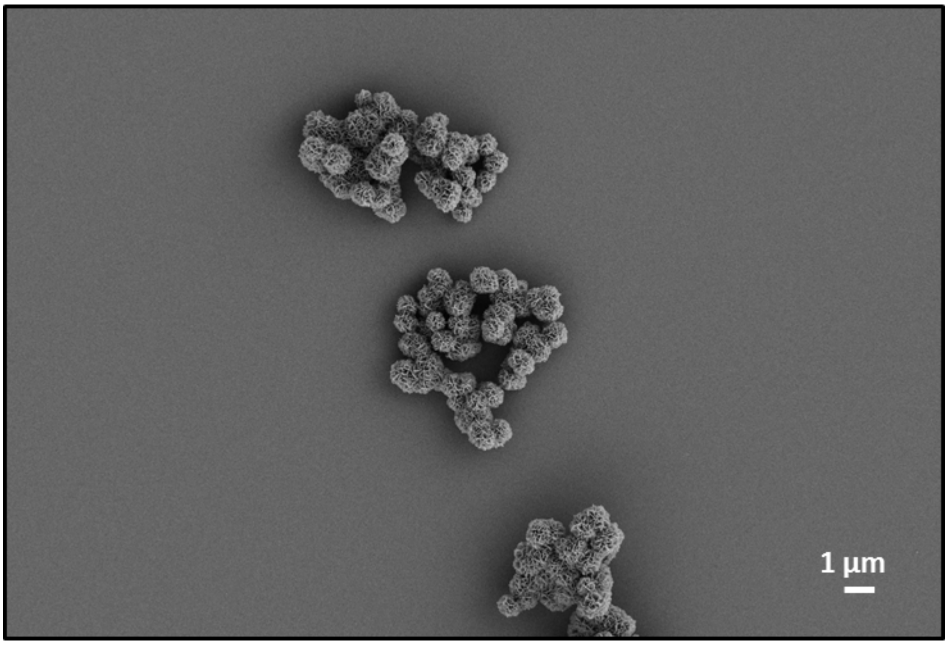

Figure S44. SEM image used to measure the size distribution of the particles generated from cyclic **1**_PO_4. The RCA was carried out for 20 h in the presence of 25 mM Mg^{2+} using Φ-29 DNA polymerase. ImageJ was used to measure the average diameter of particles: 0.89 ± 0.15 μm, n = 20 (n refers to the number of measured particles).

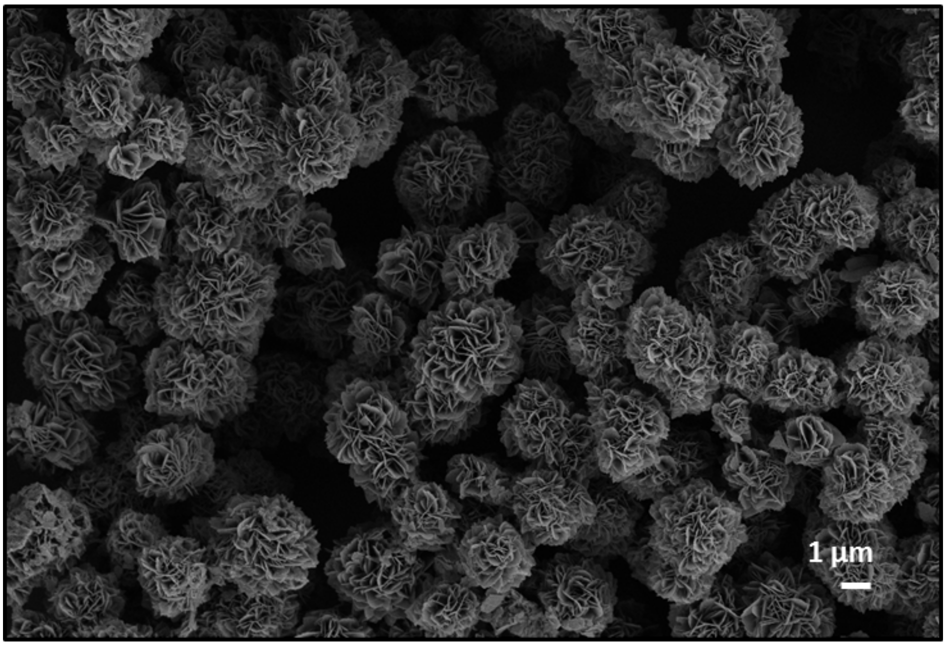

Figure S45. SEM image used to measure the size distribution of the particles generated from cyclic **1**_PA. The RCA was carried out for 20 h in the presence of 10 mM Mg^{2+} using Φ-29 DNA polymerase. ImageJ was used to measure the average diameter of particles: 3.40 ± 0.35 μm, n = 23 (n refers to the number of measured particles).

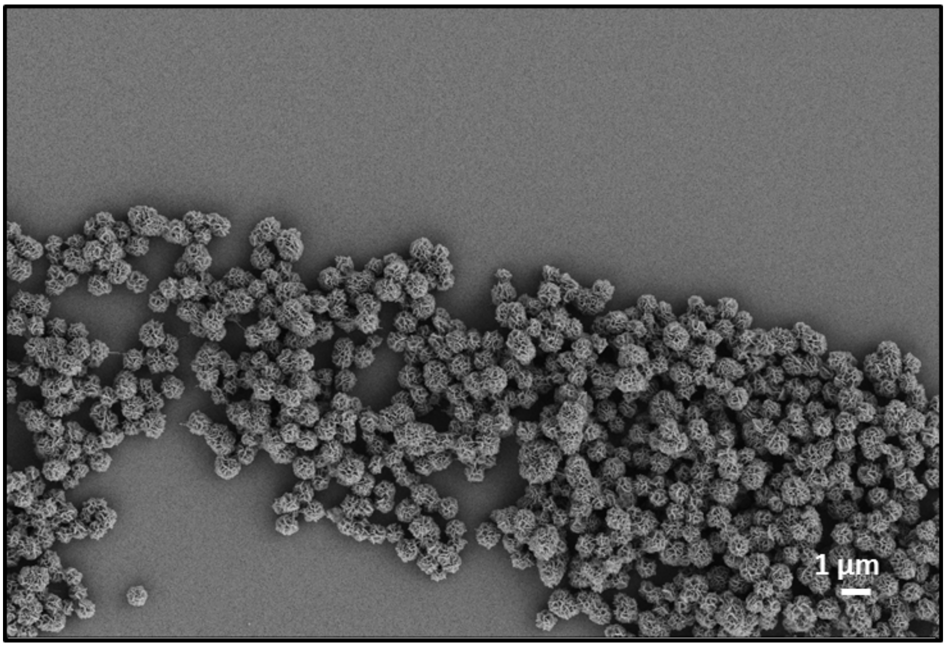

Figure S46. SEM image used to measure the size distribution of the particles generated from cyclic **1**_PA. The RCA was carried out for 20 h in the presence of 25 mM Mg^{2+} using Φ-29 DNA polymerase. ImageJ was used to measure the average diameter of particles: 0.77 ± 0.07 μm, n = 60 (n refers to the number of measured particles).

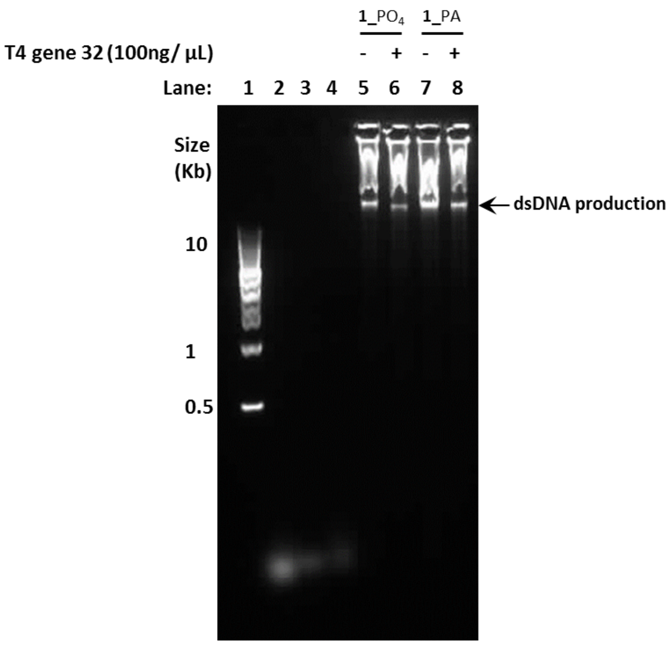

Figure S47. 0.8% agarose gel analysis of the reaction mixtures of RCA reactions performed in the presence of single-stranded binding protein. The RCA reactions were performed in the presence of 10 mM Mg^{2+} using the cyclic templates **1**_PO_4 and **1**_PA and Φ-29 DNA polymerase. Lane 1: 1 kb DNA ladder; Lane 2: splint (ODN 15); Lane 3: cyclic unmodified template (**1**_PO_4); Lane 4: cyclic phosphoramidate template (**1**_PA). Arrow indicate the band which is assigned to double-stranded DNA formation during RCA process, by analogy with a previous literature report.(4) We found that the T4 gene 32 protein could not reduce dsDNA production for our cyclic templates.

References

1. El-Sagheer, A.H., Sanzone, A.P., Gao, R., Tavassoli, A. and Brown, T. (2011) Biocompatible artificial DNA linker that is read through by DNA polymerases and is functional in Escherichia coli. *Proc. Natl. Acad. Sci.*, **108**, 11338-11343.
2. Shivalingam, A., Tyburn, A.E.S., El-Sagheer, A.H. and Brown, T. (2017) Molecular Requirements of High-Fidelity Replication-Competent DNA Backbones for Orthogonal Chemical Ligation. *J. Am. Chem. Soc.*, **139**, 1575-1583.
3. Zuker, M. (2003) Mfold web server for nucleic acid folding and hybridization prediction. *Nucleic Acids Res.*, **31**, 3406-3415.
4. Ducani, C., Bernardinelli, G. and Högberg, B. (2014) Rolling circle replication requires single-stranded DNA binding protein to avoid termination and production of double-stranded DNA. *Nucleic Acids Res.*, **42**, 10596-10604.
